# An oncolytic adenovirus targeting *SLAMF7* demonstrates anti-myeloma efficacy

**DOI:** 10.1038/s41375-025-02617-3

**Published:** 2025-04-17

**Authors:** Georgia Stewart, Simon Tazzyman, Yidan Sun, Rebecca E. Andrews, Jack Harrison, Darren Lath, Jenny Down, Georgia Robinson, Xue Wang, Munitta Muthana, Andrew. D. Chantry, Michelle A. Lawson

**Affiliations:** 1 Sheffield Myeloma Research Team, University of Sheffield, Sheffield, UK; 2 Mellanby Centre for Musculoskeletal Research, University of Sheffield, Sheffield, UK; 3 Division of Clinical Medicine, School of Medicine and Population Health, University of Sheffield, Sheffield, UK; 4 Department of Haematology, Sheffield Teaching Hospitals NHS Foundation Trust, Royal Hallamshire Hospital, Sheffield, UK

**Keywords:** Targeted therapies, Cancer models

## Abstract

We investigated a novel *SLAMF7*-promoter driven oncolytic adenovirus (Ad[CE1A]) as a potential therapeutic for multiple myeloma, an incurable hematological malignancy. Ad[CE1A] infection, replication, and oncolysis were assessed in a panel of myeloma cell lines (n = 8) and ex vivo samples from myeloma patients (n = 17) and healthy donors (HDs) (n = 14). Ad[CE1A] efficiently infected, replicated, and induced oncolysis in myeloma cells, but not in control cell lines or HDs, demonstrating selective cytotoxicity. Mechanistic studies revealed Ad[CE1A]-induced cell death is caspase-independent, with a potential involvement of necroptosis. Ad[CE1A] also altered immunogenic cell death markers (calreticulin, CD47, extracellular ATP), enhanced antigen presentation via increased MHC class I and II receptor expression (HLA-ABC and HLA-DR), and stimulated bystander cytokine killing, indicating potential for direct and immune-mediated anti-myeloma responses. In vivo experiments with 5TGM1 syngeneic and U266 xenograft models showed Ad[CE1A] significantly reduced myeloma tumor burden compared to vehicle control. Combination therapy with anti-myeloma drugs, bortezomib, melphalan, panobinostat and pomalidomide, enhanced Ad[CE1A] efficacy, with melphalan upregulating *SLAMF7*, resulting in increased viral replication. In summary, these findings support Ad[CE1A] as a promising myeloma therapy.

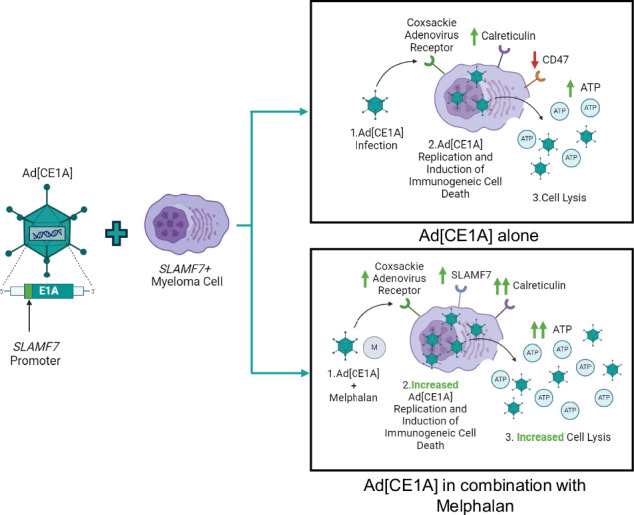

## Introduction

Multiple myeloma is a genetically and clinically heterogeneous hematological malignancy of plasma cells, which proliferate primarily in the bone marrow. Myeloma clinical manifestations include bone disease, anemia, renal impairment and immunodeficiency [1]. Worldwide, ~176,000 people are diagnosed with myeloma each year [2], with incidence expected to increase with an ever aging population [3, 4]. Whilst recent treatment advancements have improved the five-year survival rate to 52.3% [3], myeloma remains incurable, necessitating the need for new and safe treatments.

Novel therapies not reliant on generic chemotherapies and their associated toxicities are desired. Oncolytic viruses (OVs) are an emerging form of anti-cancer therapy that treat cancer with replicating viruses, inducing direct cell death and engagement of both the innate and adaptive anti-tumor immune responses via the release of inflammatory cytokines, tumor-associated antigens (TAAs), and danger-associated/pathogen-associated molecular patterns (DAMPs/PAMPs) [5]. OVs released after lysis can spread to local or distant tumor sites, amplifying their effects [6], and can synergize with cancer therapies to overcome drug resistance [6].

Adenovirus serotype 5 (Ad5) is a double-stranded DNA virus [7] that uses the coxsackie adenovirus receptor (CAR) [8] and αvβ_3/5_ integrins for viral entry [9]. Ad5 is amongst the most extensively studied OV [10], has demonstrated a good safety profile after two decades of clinical trials, and has also shown anticancer efficacy [10, 11]. Tumor-specific Ad5s have been developed, in which the promoters of cancer-related genes are used to regulate virus replication [12, 13]. Ad5 replication is reliant on transcription of early region 1A (*E1A*), and therefore control of *E1A* by a tumor-specific promoter largely restricts replication to cancer cells. Promoters for established tumor markers have previously been used, such as hTERT [13, 14], E2F [15] and prostate-specific antigen (PSA) [12]. Since the signaling lymphocytic activation molecule family member 7 (*SLAMF7*) gene is highly and consistently upregulated across all stages of myeloma [16], and is the target antigen (CS1) of the monoclonal antibody Elotuzumab [17], *SLAMF7* was therefore chosen as it would potentially make a stable target for a tumor-specific promoter for an oncolytic Ad5. *SLAMF7* was also chosen as a target over other myeloma markers, such as B-cell maturation antigen (BCMA) and CD38, because BCMA expression can be downregulated or lost entirely in patients who have undergone BCMA-targeted therapies [18]. This downregulation of BCMA, along with the rise of BCMA-targeted therapies [19], leads to concern about a growing cohort of patients who may become resistant to BCMA-based treatments. Targeting *SLAMF7* offers an alternative that could be effective in this patient subset. While we recognize that targeting *SLAMF7* alone may not address all cases of myeloma, it represents a promising target that could complement therapies aimed at other markers. Ultimately, we believe that a multi-targeted approach incorporating *SLAMF7*-targeted therapies could enhance therapeutic coverage and efficacy.

Therefore, we have developed a novel *SLAMF7*-specific replication-competent oncolytic Ad5 (Ad[CE1A]) in which the *SLAMF7* gene promoter drives the expression of the viral replication gene *E1A*. In this present study we have assessed the efficacy of Ad[CE1A] alone and/or in combination with other myeloma standard of care therapies in vitro, ex vivo, and in vivo.

## Materials and methods

### Ethics statement

All methods were performed in accordance with the relevant guidelines and regulations. Human samples were collected from Sheffield Royal Hallamshire Hospital with ethical approval (REC reference:05/Q2305/96) following written and informed consent. Animal procedures were approved by the UK Home Office (PPL: PP1099883) and the University of Sheffield’s Animal Ethics Committee. Mice were housed in ventilated cages with constant access to standard diet and tap water, in a temperature and humidity-controlled room on a 12-hour light/dark cycle.

### Cell culture

All cell lines (Supplementary Table 1) and culture conditions are described in Supplementary Data.

### Patient-derived primary Cells

Peripheral blood and bone marrow (BM) samples were collected from myeloma patients, monoclonal gammopathy of undetermined significance (MGUS) patients, plasma cell leukemia (PCL) patients and healthy donors (HDs) (Supplementary Table 2). Primary cell culture and CD138 positive (^+^) and negative (^-^) isolation is described in Supplementary.

### Viruses and inhibitors

The following viruses were used: Ad[CE1A], a conditionally replicating *SLAMF7-*promoter driven Ad5 (see Supplementary and Supplementary Fig. 1); Ad-GFP, a recombinant non-replicative E1A/E1B deleted human Ad5 virus expressing GFP under CMV promoter; Ad[PSA], a conditionally replicating oncolytic Ad5, whose replication is restricted by prostate-specific promoter elements [12]. All inhibitors/drugs were purchased from Selleckchem (Cambridge, UK).

### Assessment of adenoviral virion production

1 × 10^6^ cells were infected with Ad[CE1A] at a multiplicity of infection (MOI) 2 or with PBS control. After 24 h media was replaced, and after 72 h viral titer was determined using the Adeno-X^TM^ rapid titer kit, as per manufacturer’s instructions (Clontech, London, UK).

### Flow cytometry

Flow cytometry was performed using a FACSCalibur (Becton Dickinson, Oxford, UK) or LSR II (BD Biosciences, Oxford, UK), data were analyzed using the FlowJo^TM^ software (v.10.5.0) (FlowJo LLC, Oregon, USA). Antibody details are provided in Supplementary Table 3. For staining protocols see Supplementary Methods.

### Cell viability and death assays

For cell viability assays, 1 × 10^4^ cells were seeded in 100 µL complete RPMI media. For cell death assays, 1 × 10^5^ cells were seeded in 0.5 mL complete RPMI media. Cells were treated with vehicle, Ad[CE1A] ± apoptosis/necrosis inhibitors, or anti-myeloma therapies at indicated suboptimal doses. AlamarBlue® reagent (Thermo Fisher Scientific) was added between 24–96 h following manufacturer’s instructions. Cytotoxicity was assessed by propidium iodide using flow cytometry.

### RT-qPCR

Total RNA was extracted from 2 × 10^6^ cells (cell lines) or 5 × 10^5^ cells (primary cells) at specified time points, after treatment with vehicle or Ad[CE1A] ± anti-myeloma therapies at indicated doses, using the ReliaPrep™ RNA Miniprep Systems kit (Promega, UK). RNA was reverse transcribed using a High-Capacity cDNA to RNA kit (Applied Biosystems, UK), followed by Taqman^TM^ or SYBR® green gene expression RT-qPCR using primers listed in Supplementary Table 4. For human cells, qPCR was normalized to GAPDH, for mouse cells, qPCR was normalized to B2M. Relative quantification was performed using the 2^^^(-delta delta CT) method.

### Enlighten® ATP assay

1 × 10^5^ cells were treated with either vehicle or Ad[CE1A] ± anti-myeloma therapies. After 24 h cell-free supernatant was collected and ATP concentration assessed using the Enlighten® ATP assay (Promega) as per manufacturer’s instructions.

### Animal studies

Both a moderately aggressive xenograft model using U266 cells and an aggressive murine syngeneic myeloma model using 5TGM1 cells were used. In NSG and C57BL/KaLw/RijHSD mice, these cell types specifically colonize the bone after intravenous (i.v) injection described previously [20]. In both studies, mouse numbers were calculated using power calculations using G*Power software, where α (significance level) was 0.05, β (power level) was 90% based on previous tumor burden data from a similar study. Confounders regarding treatment were minimized by ensuring treatment was performed by the same person, at the same time of day between 9 am and 11 am and randomizing the treatment order.

#### U266 NSG xenograft model

6–8 week old female NOD/SCID Gamma (NSG) mice (Charles River Laboratories, UK) were intravenously (i.v) injected with 1 × 10^6^ U266-GFP-Luciferase (Luc) cells via tail vein. After 5 weeks, mice were randomized based on body weight (19.25 g ± 1.62 SD) (n = 5/group, n = 10 total) into vehicle (PBS 100 µL i.v) or Ad[CE1A] (1×10^7^ ifu/100 µL i.v) groups and treated twice a week for 3 weeks, then euthanized at 8 weeks post-tumor injection. Following ex vivo tumor burden analysis, a mouse was excluded from the Ad[CE1A] treated group as it was a statistical outlier based on the Grubbs’ test (α = 0.05).

#### 5TGM1 C57BL/KaLw/RijHSD syngeneic model

6–8 week old male C57BL/KaLw/RijHSD mice (Envigo, Netherlands) were i.v injected with 2 × 10^6^ 5TGM1-Luc cells via tail vein (n = 40). After 3 days, mice were randomized based on body weight (21.7 g ± 1.14 SD) (n = 10/group) into vehicle (100 µL PBS i.v + I.P), Ad[CE1A] low dose i.v (1×10^7^ ifu/100 µL), Ad[CE1A] high dose i.v (1 × 10^8^ ifu/100 µL) or Ad[CE1A] intraperitoneal (i.p) (1 × 10^8^ ifu/100 µL) groups and treated twice a week for 4 weeks. Tumor burden was monitored bi-weekly using bioluminescent imaging (IVIS Lumina II, Caliper Life Sciences) then euthanized at 4 weeks post-tumor injection.

In all studies, at sacrifice bones were harvested to assess tumor burden, as detailed in the Supplementary. All analysis was performed blinded.

### Statistical analyses

CompuSyn® v.1.0 software generated synergy combination index (CI). GraphPad Prism v9.0 (San Diego, USA) was used to determine inhibitory dose 50 (IC_50_) values, normal distribution was assumed, and data were analyzed using either a student’s *t* test, one-way ANOVA, or two-way way ANOVA with appropriate correction methods (Tukey’s, Dunnett’s or Šidák’s) as stated in the figure legend. Significance thresholds were p < 0.05.

## Results

### Human myeloma cells are susceptible to adenovirus infection

The appeal for oncolytic Ad therapy for hematological cancers has been limited, largely due to the belief that Ad5 relies on CAR expression for infection, which is thought to be lower or absent in hematological cells compared to other cell types [21, 22]. However, the majority of human myeloma cell lines (except KMS-12-BM) do have similar frequency (%) of CAR receptor expression, although level of expression (MFI) is significantly lower in most myeloma cell lines than the HEK293A adenoviral susceptible cell line (Supplementary Fig. 2a.i, 2bi & 3a). All myeloma cells tested also express secondary receptors, αvβ_5_ and αvβ_3_ integrin, with higher αvβ_5_ expression observed than αvβ_3_ (Supplementary Fig. 2a.ii-a.iii, 2b.ii-b.iii 3b, c). This aligns with gene expression data from a publicly available dataset (GDS1067) of CAR and integrin subunits (β and α) in purified plasma cells from MGUS, myeloma, and PCL patients (Supplementary Fig. 2c), showing all three receptors expressed across these conditions. Predictably, due to CAR and αvβ_5_ expression, human myeloma cells demonstrated high infection rates, with some reaching up to 98% (time and dose dependent) (Supplementary Fig. 3d, e.ii).

### Ad[CE1A] has oncolytic activity in human myeloma cell lines

**Fig. 1:**
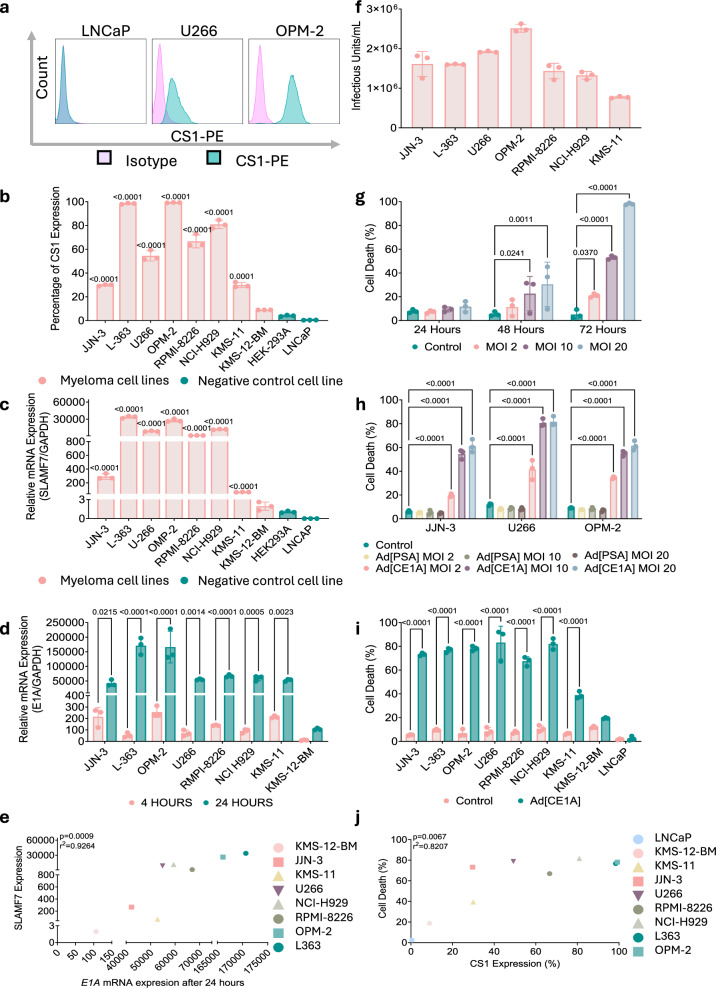
Human myeloma cell lines are susceptible to Ad[CE1A] replication and oncolysis. **a** Flow cytometry histograms of CS1 mean fluorescent intensity (MFI) in LNCaP, U266 and OMP-2 cells. **b** Percentage of CS1 expression in myeloma cells and LNCaP cells vs. HEK293A cells. **c** Relative *SLAMF7* (CS1) mRNA expression in myeloma cells and LNCaP cells vs. HEK293A cells by RT-qPCR. n = 3 biological replicates ±SD. p values: student’s *t* test vs. HEK293A cells. **d** *E1A* mRNA expression in myeloma cells at 4 and 24 h post Ad[CE1A] infection (MOI 20) compared to vehicle controls n = 3 biological replicates ±SD. p values: 2-way ANOVA Šidák’s correction. **e** Correlation of *SLAMF7* mRNA vs. *E1A* expression after 24 h of Ad[CE1A] infection (MOI 20). Correlation and p value determined by Pearson’s test. **f** Infectious virion production in myeloma cells after 72 h using Adeno-X^TM^ rapid titer kit. n = 3 biological replicates ±SD. **g** Dose and time response of Ad[CE1A] cytotoxicity in JJN-3 cells assessed by PI staining and flow cytometry. n = 3 biological replicates ±SD. p values: 2-way ANOVA Dunnett’s correction. **h** Ad[CE1A] vs. Ad[PSA] cytotoxicity (MOI 2, 10 or 20) in myeloma cells after 72 h, assessed by PI staining and flow cytometry. n = 3 biological replicates ±SD. p values: 2-way ANOVA with Dunnett’s correction. **i** Ad[CE1A] (MOI 20) cytotoxicity in myeloma cells and LNCaP cells after 72 h assessed by PI staining and flow cytometry. n = 3 biological replicates ±SD. p values: 2-way ANOVA Šidák’s correction. **j** Correlation of CS1 surface expression vs. cell death after 72 h Ad[CE1A] treatment (MOI 20) (average of biological triplicates plotted). Correlation and p value determined by Pearson’s test.

Ad[CE1A] utilizes CS1 (*SLAMF7*) for transcriptional control of the essential replication gene E1A. Most human myeloma cell lines tested exhibited higher CS1 cell surface and/or *SLAMF7* mRNA expression compared to HEK293A cells (Fig. 1a–c). LNCaP prostate cancer cells did not express CS1 at gene or protein level. Ad[CE1A]’s replication was subsequently investigated. Firstly, *E1A* expression increased over time in the majority of myeloma cells (Fig. 1d), which showed a strong positive correlation with *SLAMF7* expression (p < 0.0009 and an R^2^ of 0.9264) (Fig. 1e). Secondly, all myeloma cell lines tested produced infectious viral progeny (Fig. 1f).

Once infection and replication of Ad[CE1A] was established, its oncolytic activity was assessed. Ad[CE1A] significantly induced myeloma cell death dose-dependently (Fig. 1g). To attribute cell death to Ad[CE1A] replication via the *SLAMF7* promoter and not just to viral infection/load, Ad[PSA] was used as a replicative control. Only Ad[CE1A] significantly increased myeloma cell death (Fig. 1h), not Ad[PSA], providing evidence that cell death is due to *SLAMF7-*replication and not initial viral infection/load. Further assessment was extended to more myeloma cell lines, which showed significant cell death in most myeloma cell lines, whilst CS1-negative LNCaP cells were not affected (Fig. 1i). Mean cell death positively correlated with CS1 surface expression (p < 0.0067 and an R^2^ of 0.8207) (Fig. 1j), giving further evidence of Ad[CE1A]’s specificity to CS1 expressing cells.

### Primary patient-derived myeloma cells are susceptible to Ad[CE1A] infection and oncolysis

**Fig. 2:**
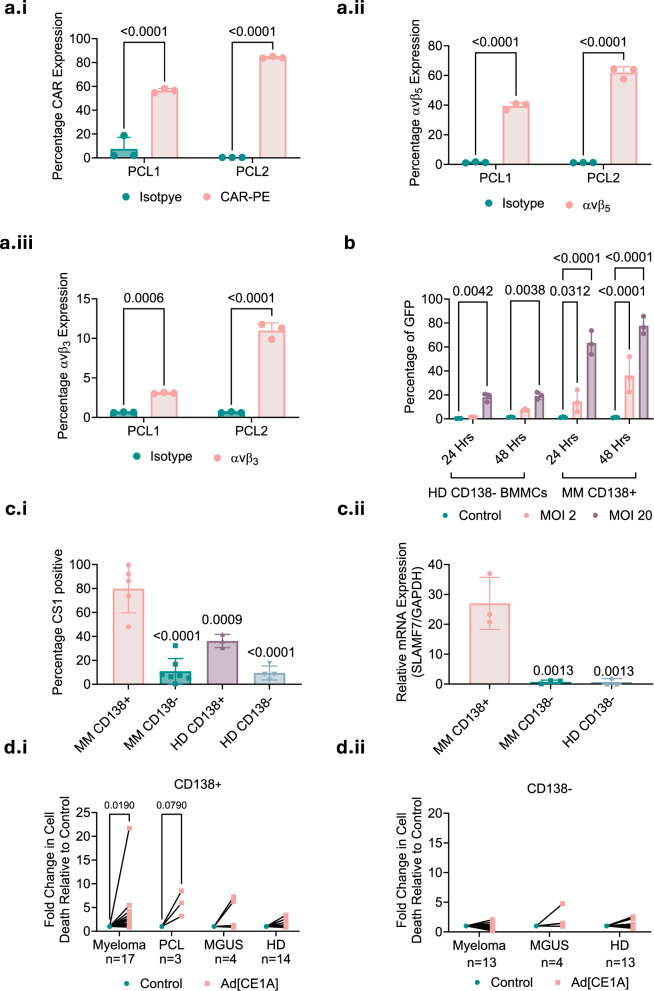
Primary patient derived myeloma cells are susceptible to Ad[CE1A] infection and oncolysis. Percentage expression of (**a.i**) coxsackie adenovirus receptor (CAR), (**a.ii**) αvβ_5_ and (**a.iii**) αvβ_3_ in primary patient-derived plasma cell leukemia cells PCL1 and PCL2 cells vs. dose-matched isotype controls assessed by flow cytometry. n = 3 biological replicates ±SD. p values: two-way ANOVA Šidák’s correction. **b** Percentage GFP expression in CD138^+^ myeloma (MM) cells and healthy donors (HD) CD138^-^ bone marrow mononuclear cells (BMMCs) after 24 and 48 h of Ad-GFP infection (MOI 2 or 20). n = 3 biological replicates ±SD. p values: 2-way ANOVA Dunnett’s correction. **c.i** Percentage CS1 surface expression assessed by flow cytometry and **c.ii** relative *SLAMF7* expression assessed by RT-qPCR in CD138^+^ and CD138^-^ populations from MM patients and HD. ±SD. p values: one-way ANOVA Dunnett’s correction. **d.i** Relative Ad[CE1A] (MOI 20) cytotoxicity compared to vehicle control after 96 h in MM (n = 17), plasma cell leukemia (PCL) (n = 3), monoclonal gammopathy of undetermined significance (MGUS) (n = 4) and HD CD138^+^ cells (n = 14). **d.ii** Relative Ad[CE1A] (MOI 20) cytotoxicity compared to control after 96 h in MM (n = 13), MGUS (n = 4) and HD (n = 13) CD138^-^ cells. p values: paired T test.

To evaluate Ad[CE1A]’s effect on primary cells, we first assessed Ad5 receptor expression in patient-derived PCL cells (PCL1and PCL2), which showed comparable expression to myeloma cell lines (Fig. 2a.i-iii). The infection rate in CD138^+^ myeloma patient-derived plasma cells was also comparable to myeloma cells (Fig. 2b) and was significantly greater than in the HD CD138^-^ bone marrow mononuclear cell (BMMC) population, additionally Ad-GFP had no significant effect on viability in these populations after 48 h, evidencing that initial viral infection/load alone does not influence viability (Supplementary Fig. 3f). CD138^+^ myeloma patient-derived plasma cells also displayed significantly higher CS1/*SLAMF7* expression than myeloma CD138^-^ BMMCs, HD CD138^+^ plasma cells and HD CD138^-^ BMMCs. (Fig. 2c.i-ii). Crucially, when Ad[CE1A] efficacy in primary myeloma cells was investigated, myeloma and PCL CD138^+^ cells showed significant cell death vs. vehicle control (Fig. 2d.i, Supplementary Fig. 4a.i). There was a trend for increased cell death in the CD138^+^ MGUS following Ad[CE1A] therapy with some patients responding to Ad[CE1A], but the n numbers were low in this group (n = 4). Importantly, the cells were cultured in 10% autologous serum, which potentially contained neutralizing anti-huAd5 antibodies (not tested), which highlights Ad[CE1A]’s potential ability to induce cell death in conditions where neutralizing anti-huAd5 antibodies may be present. Variable cell death levels were observed, potentially due to patient heterogeneity, or neutralizing antibodies. Importantly, Ad[CE1A] did not cause any significant cell death in pre-malignant CD138^+^ HD cells (Fig. 2d.i, Supplementary Fig. 4a.i) or in any CD138^-^ population from myeloma, MGUS and HD patients (Fig. 2d.ii, Supplementary Fig. 4a.ii). To assess ‘off-target’ effects of Ad[CE1A] on immune subsets known to express *SLAMF7*, such as NK cells, CD8 T cell and CD4 T cells, 3 patient samples were assessed. No significant reduction in these immune subsets, even at an elevated MOI of 30 (Supplementary Fig. 4b.i-d.iii) were observed. Taken together these findings are encouraging and suggest Ad[CE1A] has limited ‘off-target’ effects, supporting its potential therapeutic value.

### Ad[CE1A] induces caspase-independent cell death, potential role of necroptosis

**Fig. 3:**
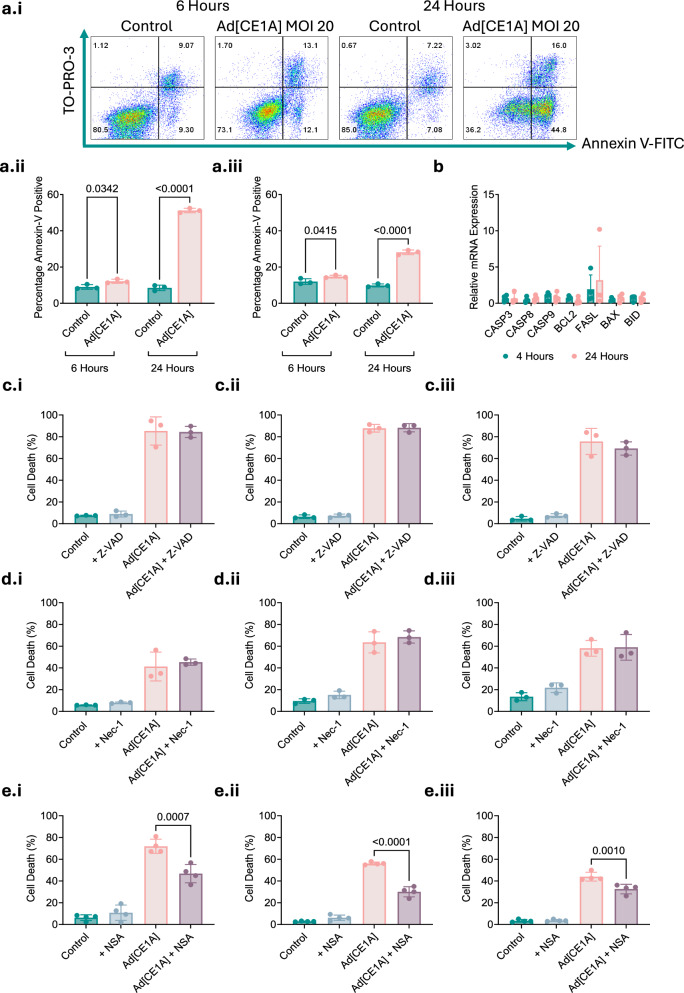
Ad[CE1A] induces caspase independent cell death whilst the necroptosis inhibitor of MLKL inhibits Ad[CE1A] induced cell death. **a.i** Representative scatter plots: Annexin V vs. TO-PRO-3 in JJN-3 cells after Ad[CE1A] MOI 20 at 6 and 24 h. Percentage Annexin V-positive cells in (**a.ii**) JJN-3 and (**a.iii**) U266 cells after Ad[CE1A] MOI 20 after 6 and 24 h. n = 3 biological replicates ±SD. p values: 2-way ANOVA Šidák’s correction. **b** Apoptotic gene expression (caspase 3/8/9, BCL2, FASL, BAX and BID) at 6 and 24 h post Ad[CE1A] (MOI 20) treatment. n = 4 biological replicates ±SD. p values: 2-way ANOVA Šidák’s correction. **c.i**–**iii** Cell death after 72 h with Ad[E1A] MOI 10 ± pan caspase inhibitor (50 µM Z-VAD-FMK) in JJN-3, U266 and OPM-2 cells, n = 3 biological replicates. **d.i**–**iii** Cell death after 72 h with Ad[E1A] MOI 10 ± RIPK1 inhibitor (50 µM Nec-1) in JJN-3, U266 and OPM-2 cells, n = 3 biological replicates. **e.i**–**iii** Cell death after 72 h with Ad[E1A] MOI 10 ± MLKL inhibitor (5 µM NSA) in JJN-3, U266 and OPM-2 cells. n = 4 biological replicates ±SD. p values: one-way ANOVA Tukey’s correction.

Oncolytic Ad5’s mechanism of cell death remains unclear, so we investigated whether Ad[CE1A] induces apoptosis. There was a significant increase in Annexin V^+^ TO-PRO-3^−^ expression after 24 h of Ad[CE1A] treatment (Fig. 3a.i-a.iii). In contrast, apoptotic genes showed no consistent upregulation (Fig. 3b), and caspase inhibition (Z-VAD-FMK- pan caspase inhibitor) did not prevent Ad[CE1A]-induced cell death (Fig. 3c.i–iii). Interestingly, phosphatidylserine (PS) exposure (target of Annexin V), can occur in non-apoptotic forms of regulated inflammatory cell death, such as necroptosis [23].

Given these results, we next assessed Ad[CE1A] effects on necroptosis. Necroptosis, caspase-independent regulated cell death that manifests a necrotic morphotype, is controlled by receptor-interacting proteins 1 (RIPK1) and 3 (RIPK3) and mixed lineage kinase domain-like protein (MLKL) [24]. Pharmacological inhibition of RIPK1 (Necrostatin-1 (Nec-1)) (Fig. 3.d.i-d.iii) and RIPK3 (GSK-872) (data not shown) failed to protect myeloma cells from Ad[CE1A]-induced cell death. However, inhibition of MLKL with necrosulfonamide (NSA) did significantly protect myeloma cells from Ad[CE1A]-induced cell death (Fig. 3.e.i–iii). However, complete protection was not achieved, suggesting involvement of other mechanisms.

### Ad[CE1A] induces the expression of immunogenic cell death markers, MHC class I and II and bystander cytokine killing

**Fig. 4:**
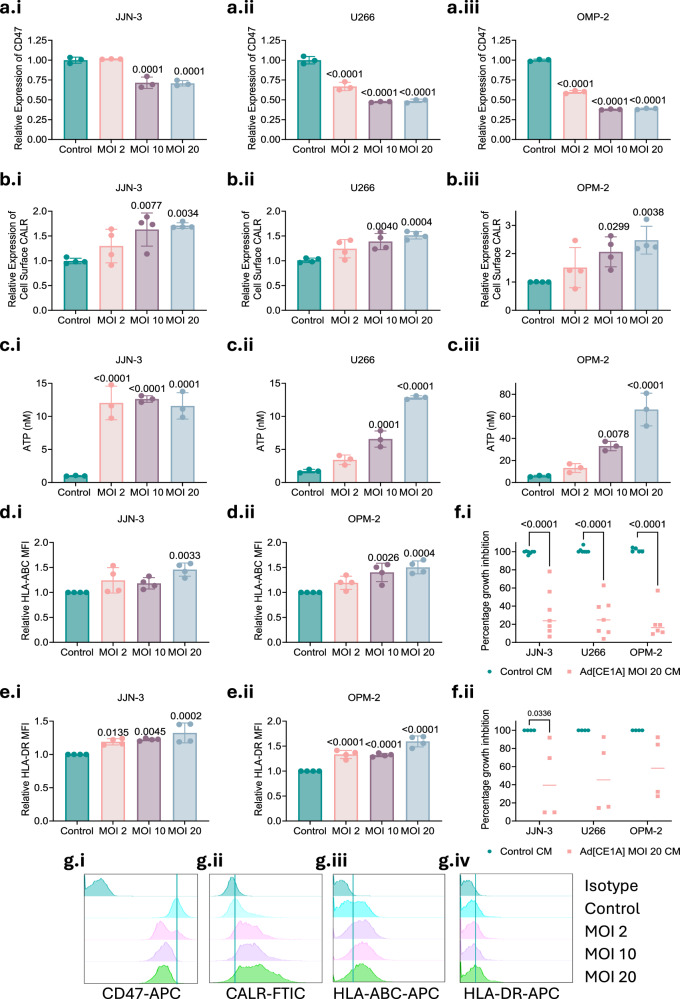
Ad[CE1A] modulates CD47, CALR, ATP, MHC Class I and II expression, and induces cytokine-mediated myeloma cell killing. JJN-3, U266 and OPM-2 cells were treated with Ad[CE1A] (MOI 2, 10 or 20) or vehicle control. After 24 or 48 h, viable cells were assessed for changes in CD47 or CALR expression by flow cytometry. **a.i**–**iii** Relative MFI of CD47 was compared to vehicle control in JJN-3, U266 and OPM-2 cells, n = 3 biological replicates. **b.i**–**iii** Relative MFI of CALR was compared to vehicle control in JJN-3, U266 and OPM-2 cells, n = 4 biological replicates. **c.i**–**iii** Extracellular release of ATP in myeloma cells following Ad[CE1A] (MOI 2, 10, or 20) treatment in JJN-3 U266 and OPM-2 cells after 24 h. ATP concentration was determined using the ENLITEN® ATP assay using an ATP standard curve, n = 3 biological replicates. JJN-3, U266 and OPM-2 cells were treated with Ad[CE1A] (MOI 2, 10 or 20) or vehicle control. After 48 h, viable cells were assessed for changes in HLA-ABC or HLA-DR. Relative MFI of HLA-ABC (**d.i**–**ii**) or HLA-DR (**e.i**, **e.ii**) was compared to vehicle control in JJN-3 and OPM-2 cells, n = 3 biological replicates. ±SD. p values: one-way ANOVA Dunnett’s correction. JJN-3, U266 and OPM-2 cells were cultured in UV-inactivated CM (1:1) from BMMCs from (**f.i**) HDs (n = 7) or (**f.ii**) MM patients (n = 4) exposed to Ad[CE1A] for 48 h. MPC viability determined after 96 h using AlamarBlue® assay. p values: 2-way ANOVA Šidák’s correction. Representative histogram plots of viable OPM-2 cells after Ad[CE1A] treatment (MOI 2, 10 20) assessing expression of (**g.i**) CD47 after 48 , (**g.ii**) CALR after 24 h (**g.iii**) HLA-ABC and (**g.iv**) HLA-DR after 48 h.

Since OV oncolysis can be highly immunogenic [25] we assessed DAMP-associated immunogenic cell death (ICD) markers (CD47, CALR and ATP). CD47, an anti-phagocytic molecule, significantly decreased after 24 (data not shown) and 48 h (Fig. 4a.i–iii & g.i) after Ad[CE1A] treatment. CALR, a pro-phagocytic molecule (Fig. 4b.i-b.iii & g.ii), and extracellular ATP (Fig. 4c.i–iii), significantly increased after 24 h following Ad[CE1A] treatment. Next, since upregulation of MHC class I and II in myeloma cells could potentially aid antigen presentation and subsequent T cell activation [26] we assessed MHC expression. After 48 h, Ad[CE1A] induced a dose-response increase of MHC-Class I HLA-ABC and MHC Class II HLA-DR in all myeloma cell lines tested (Fig. 4d.i–ii, e.i–ii & g.iii–iv). Given Ad[CE1A]’s induction of ICD markers and increased MHC expression in myeloma cells, we next assessed if Ad[CE1A] could induce BMMCs to release cytotoxic cytokines resulting in bystander cytokine killing. After 96 h, Ad[CE1A]-treated HD BMMC-conditioned media (CM) significantly reduced myeloma cell viability (Fig. 4f.i). Ad[CE1A]-treated myeloma patient BMMC-CM significantly reduced viability in JJN-3 cells, but not in U266 or OPM-2 cells (Fig. 4f.ii). This variation in efficacy was likely due to differential immunogenic responses among patients. Collectively these findings suggest Ad[CE1A] can potentially modulate immune mechanisms to enhance tumor oncolysis.

### Ad[CE1A] displays in vivo efficacy in xenograft and immunocompetent murine myeloma models

**Fig. 5:**
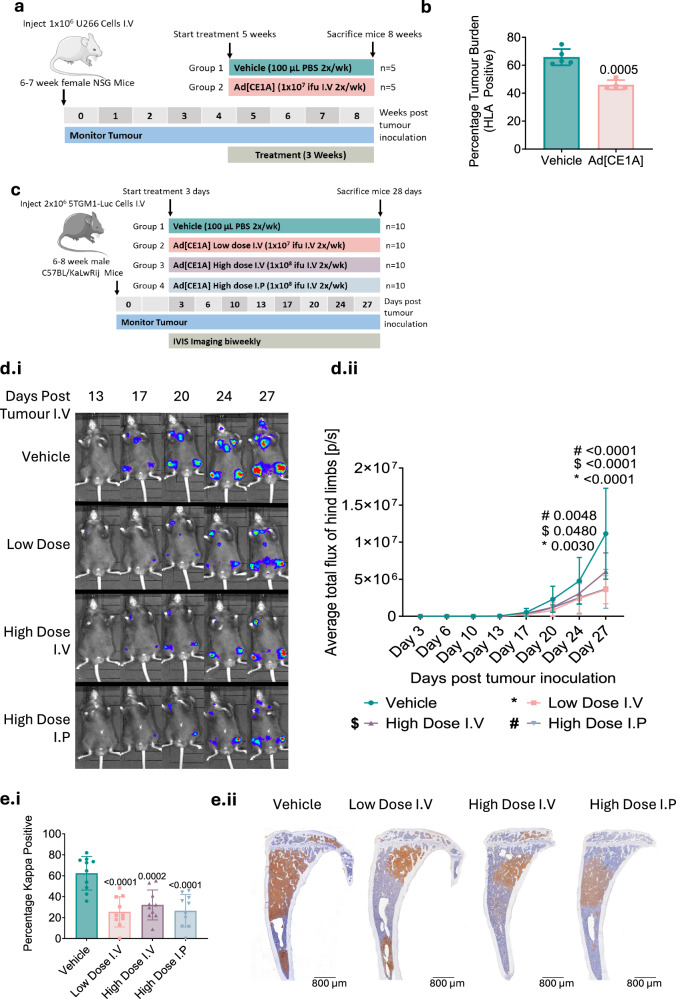
Ad[CE1A] reduces tumor burden in murine myeloma models. **a** Xenograft experimental plan: Female NSG mice were inoculated with 1 × 10^6^ U266 (I.V). 5 weeks after tumor development mice were randomized into vehicle (PBS) (n = 5) or Ad[CE1A] (10^7^ ifu) (n = 4) (I.V) 2x/week for 3 weeks. After 3 weeks, mice were euthanised. **b** Flow cytometric analysis of the tumor burden in the bone marrow using anti-human HLA-FITC antibody staining. ±SD, p values: student’s *t* test. **c** Syngeneic experimental plan: Male C57BL/KaLwRij mice were inoculated with 2 × 10^6^ 5TGM1-Luc cells (I.V). After three days, mice were randomized into vehicle (PBS), 10^7^ ifu of Ad[CE1A] I.V (low dose group), 10^8^ ifu of Ad[CE1A] I.V (high dose group) and 10^8^ ifu of Ad[CE1A] I.P (n = 10/group). Treatment was administered 2x/week for 4 weeks. After 28 days, mice were euthanised. **d.i** Representative bioluminescent images. **d.ii** Average total flux of hind limbs over time. n = 10 ± SD, p values: two-way ANOVA Dunnett’s correction. **e.i** Representative IHC images of tibiae stained with anti-kappa antibody. Scale: 800 µm. **e.ii** Average percentage of kappa positive cells. n = 10 ± SD, p values: one-way ANOVA Dunnett’s correction.

Since Ad[CE1A] reduced myeloma cell viability in vitro, without significant impact on control cells, we next assessed virus efficacy in two murine models of myeloma - U266 xenograft and 5TGM1 syngeneic. Ad[CE1A] treatment significantly reduced U266 xenograft tumor burden (44 ± 1.7% vs. 65.8 ± 2.6% vehicle) at end stage (Fig. 5a, b). For the 5TGM1 model, Ad[CE1A] was first tested in vitro (Supplementary Fig. 5), which showed comparable replication and efficacy compared to human myeloma cell lines. In the 5TGM1 model, tumor burden was significantly reduced in all Ad[CE1A]-treated groups by day 27 compared to vehicle using bioluminescent imaging and histology (Fig. 5c, d.i–e.ii). Furthermore, Ad[CE1A] had no adverse effects on myeloma-induced bone disease (Supplementary Fig. 6).

### Combining Ad[CE1A] with anti-myeloma chemotherapies augments myeloma cell line viability

**Fig. 6:**
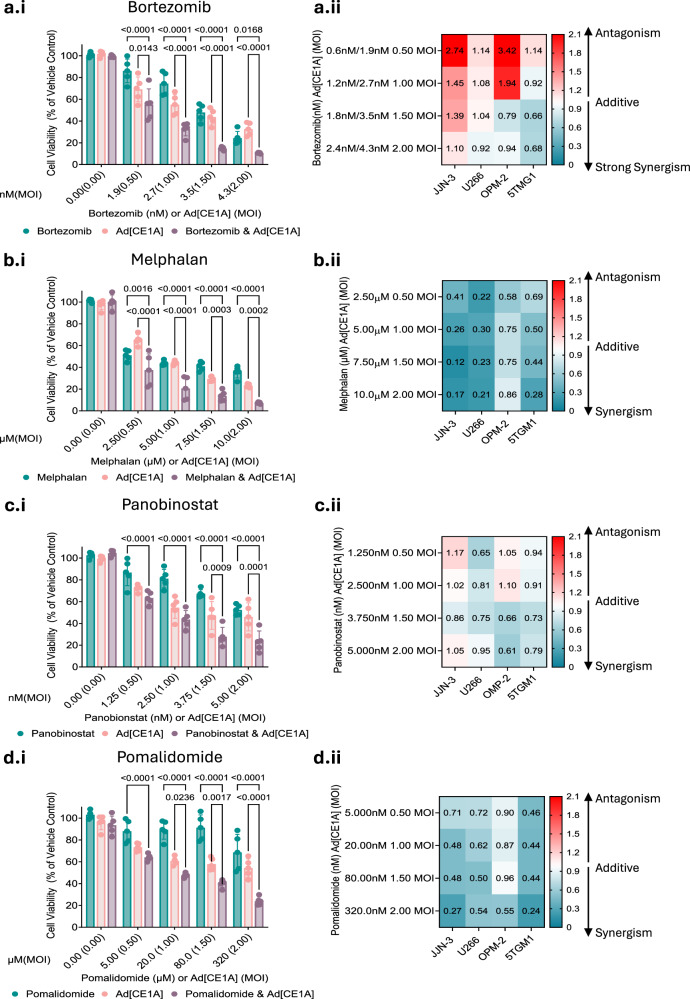
Ad[CE1A] in combination with different classes of anti-myeloma drugs results in enhanced cell death. Cell viability of Ad[CE1A] ± (**a.i**) bortezomib, (**b.i**) melphalan, (**c.i**) panobinostat or (**d.i**) pomalidomide combinations vs. monotherapies in 5TGM1 cells using AlamarBlue®. n = 5 biological replicates ±SD. p values: 2-way ANOVA Tukey’s correction. Heatmap of combination index (CI) of Ad[CE1A] in combination with (**a.ii**) bortezomib, (**b.ii**) melphalan, (**c.ii**) panobinostat or (**d.ii**) pomalidomide in human (JJN-3, U266, OPM-2) and murine (5TGM1) myeloma cell lines. For bortezomib on the y axis two doses are denoted, the first dose was used for the human myeloma cells and the second for the murine 5TGM1 cells. CI was determined by Compusyn. <0·3 strong synergism; 0·3-0·7 synergism; 0·7-0·9 moderate synergism; 0·9–1·1 additive; 1·1–1·45 slight antagonism; 1·45–2 antagonism.

Given our in vivo data and current clinical data, suggesting limited curative potential for OVs as a monotherapy [27], strategies exploring potentiating OVs or sensitizing cells to OV therapy by combinational therapies are emerging [28]. We combined Ad[CE1A] with approved anti-myeloma therapies: bortezomib, melphalan, panobinostat and pomalidomide, all with different mechanisms of action. Combination therapy augmented anti-proliferative effects in 5TGM1 cells compared to monotherapies (Fig. 6a.i, b.i, c.i, d.i), with CI analysis demonstrating synergy or additivity in human and murine myeloma cells, which is defined as a CI score of 1 or below (Fig. 6a.ii, b.ii, c.ii, d.ii). Responses to the combination therapy varied between human myeloma cell lines; this surprisingly included antagonistic effects observed at low doses of bortezomib with Ad[CE1A] (Fig. 6a.ii). Non-lethal proteasome inhibition has been shown to activate pro-tumorigenic pathways [29], this could potentially explain diminished therapeutic activity in drug combinations at low doses.

### Melphalan augments Ad[CE1A] replication

**Fig. 7:**
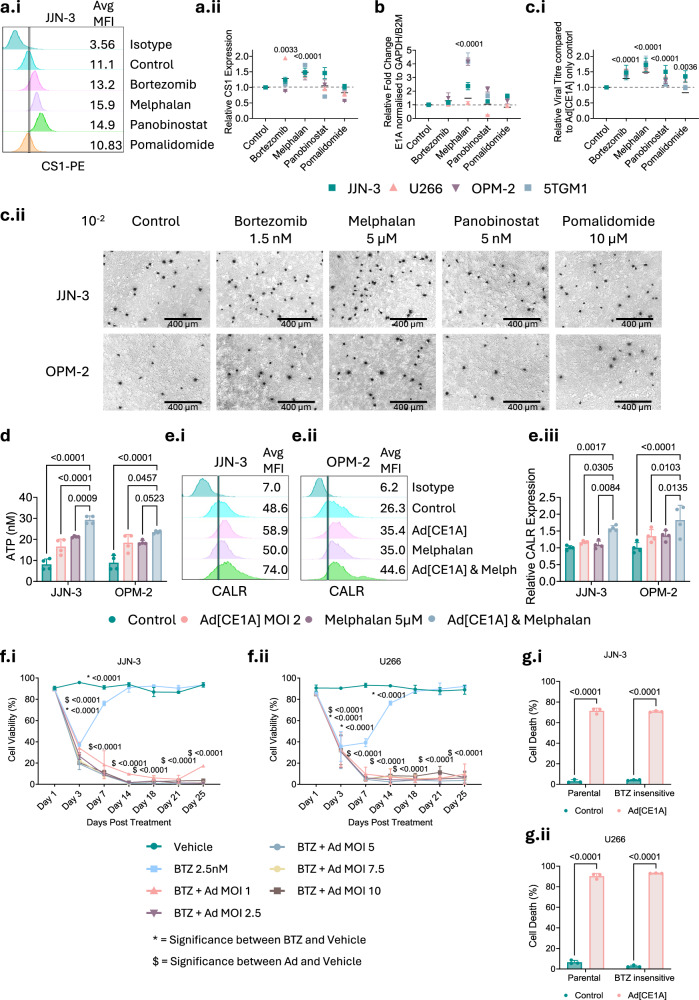
Anti-myeloma therapy augments CS1 expression, viral titer, and ICD markers and Ad[CE1A] prevents regrowth and targets bortezomib insensitive cells. **a.i** Representative histograms of CS1 MFI after 48-hour anti-MM chemotherapy in the JJN-3 cell line. **a.ii** Average relative CS1 MFI fold change after 24-hour anti-MM chemotherapy in myeloma cell lines. **b** Average *E1A* mRNA expression after 24-hour treatment with Ad[CE1A] ± anti-MM chemotherapies in myeloma cell lines. **c.i** Average relative viral titer fold change after 72-hour treatment with Ad[CE1A] ± anti-MM chemotherapies in myeloma cell lines. Black bars indicate the means of four cell lines. n = 3 biological replicates ±SD. *P* values are for one-way ANOVA Dunnett’s correction. **c.ii** Representative images of Adeno-X^TM^ rapid titer staining at 10^-2^ dilution factor. Scale bar 400 µm. **d** Extracellular ATP concentration (nM) after 24-hour treatment with Ad[CE1A] ± melph in JJN-3 and OPM-2 cells. n = 4 biological replicates, ±SD. p values: two-way ANOVA Tukey’s correction. **e** Cell surface CALR expression after 24-hour treatment with Ad[CE1A] ± melph in JJN-3 and OPM-2 cells. Representative histograms of CALR staining in (**e.i**) JJN-3 and (**e.ii**) OPM-2 cells. (**e.iii**) Relative cell surface CALR expression. n = 4 biological replicates ±SD, p values: two-way ANOVA Tukey’s correction. Cell viability of (**f.i**) JJN-3 and (**f.ii**) U266 cells after bortezomib (2.5 nM) ± Ad[CE1A] at indicated MOIs over 25 days, assessed by PI staining and flow cytometry. n = 3 biological replicates ±SD. p values: two-way ANOVA Dunnett’s correction where * denotes significance from control vs. bortezomib, $ denotes significance from control vs. Ad[CE1A]. Cytotoxicity in (**g.i**) JJN-3 and (**g.ii**) U266 parental and bortezomib insensitive cells. Percentage cell death determined by PI staining and flow cytometry. n = 3 biological replicates ±SD. p values: two-way ANOVA Šidák’s correction.

To investigate the mechanism of synergy, we first examined if the anti-myeloma therapies alter viral infection. All therapies except pomalidomide increased CAR expression; however, this did not result in increased infection (Supplementary Fig. 7). Secondly, we assessed if the anti-myeloma therapies altered viral replication. Melphalan significantly increased CS1 expression (Fig. 7a.i-a.ii), viral *E1A* expression (Fig. 7b) and viral titer (Fig. 7c). Therefore, melphalan appears to promote Ad[CE1A] replication by increasing CS1 expression. However, for the other drugs, effects on viral life cycle were less clear. Bortezomib increased CS1 expression (Fig. 7a) but not *E1A* expression (Fig. 7b). All other therapies exhibited significantly increased viral titer (Fig. 7c), despite no increase in *E1A*. Ad[CE1A] in combination with anti-myeloma therapies also increased ICD markers (data for melphalan shown in Fig. 7d-e.iii; data for bortezomib, panobinostat and pomalidomide in Supplementary Fig. 8), suggesting potential immune response enhancement.

### Ad[CE1A] is effective against bortezomib insensitive cells and prevents cell regrowth after bortezomib treatment

Myeloma typically becomes resistant or refractory to therapy, especially bortezomib [30], which is a first-line proteasome inhibitor; therefore, it was important to assess whether Ad[CE1A] can prevent regrowth after bortezomib treatment, to determine if Ad[CE1A] could potentially prevent relapse in patients or mouse models. Ad[CE1A] treatment post bortezomib treatment in vitro prevented JJN-3 and U266 cell regrowth over 25 days vs. bortezomib monotherapy, where cell viability recovered to similar levels of the vehicle controls over 14 days (Fig. 7f.i–ii). We then investigated Ad[CE1A] efficacy against bortezomib-insensitive cells (Fig. 7g.i–ii, Supplementary Fig. 9) and observed that Ad[CE1A] killed bortezomib insensitive and parental myeloma cells equally. Therefore, Ad[CE1A] is a potential treatment for patients with refractory disease.

## Discussion

Currently myeloma lacks curative therapies, necessitating the development of new and novel treatments. Previous studies have demonstrated myeloma susceptibility to a few genetically modified oncolytic Ads, both in vitro and in vivo [31]. Expanding on previous work, a novel *SLAMF7*-promoter driven oncolytic Ad5 (Ad[CE1A]), specifically targeting *SLAMF7* expressing cells, was generated and its efficacy against myeloma (cell lines, patient-derived cells and murine models) was assessed.

Firstly, we confirmed Ad5 infection of myeloma cells through CAR and αvβ_3/5_. This is controversial due to low CAR expression in hematological cells, including myeloma [21, 32, 33], but significant CAR expression has been observed in human myeloma cells before [34]. Secondly, Ad[CE1A] replication in human myeloma cells was demonstrated via *E1A* expression and infectious progeny production, correlating strongly with *SLAMF7* expression, which evidences *SLAMF7* promoter control of *E1A* expression. Thirdly, Ad[CE1A] induced significant cell death in human cell lines and, crucially, in patient-derived myeloma cells compared to vehicle controls. Notably, this effect was absent in control cell lines (LNCaP), healthy plasma cells from HD and CD138^-^ BMMC populations. Cell death positively correlated with CS1 expression, substantiating CS1-promoter-driven replication as the cause of Ad[CE1A] oncolysis. Further evidence suggested replication, not initial infection, was the cause of cell death, as Ad[PSA] had no impact on myeloma cell death. Whilst *SLAMF7* is upregulated in myeloma, it is expressed in other cell types such as NK cells, NK-like T cells and CD8^+^ T cells. In this manuscript we have shown Ad[CE1A] does not have any detrimental effects on myeloma patient T or NK cell numbers, but further detailed analyses would be required to determine if Ad[CE1A] treatment can trigger a beneficial immune response.

Myeloma cell death levels varied in patient cells, and some showed no response; this potentially highlights myeloma heterogeneity, possibly due to varying CS1 expression. In addition, Ad5 infection is prevalent in humans, so the percentage of neutralizing antibodies in the general population is high [35], potentially limiting oncolytic Ad5 clinical efficacy [36]. Whilst Ad[CE1A]-neutralization is possible, myeloma patients often have depressed antibody titers due to compromised B cell function and hypogammaglobulinemia [37], making clearance of Ad[CE1A] via neutralizing antibodies unlikely. Promisingly, ex vivo patient samples were cultured in 10% autologous serum, which potentially contained neutralizing anti-huAd5 antibodies (not tested), highlighting Ad[CE1A]’s potential ability to induce cell death in conditions where neutralizing anti-huAd5 antibodies may be present. Encouragingly, the *SLAMF7*-promoter modification has not compromised Ad5’s oncolytic ability, consistent with other modified Ad5s [21, 38, 39]. Together these findings support Ad[CE1A]’s potential clinical translation. There was a trend for Ad[CE1A]-induced cell death in CD138^+^ cells from MGUS patients, with some patients responding whilst others did not, however the n numbers for this group are small (n = 4). One possible explanation for this outcome, other than the what is discussed above, despite reports of elevated *SLAMF7* mRNA expression across monoclonal gammopathies, including MGUS [40], may be that premalignant cells retain functional antiviral defences that inhibit effective OV replication. For instance, MGUS cells are more likely to activate an interferon response upon detecting viral infection, initiating antiviral defenses that effectively restrict Ad[CE1A] replication and spread [41].

Oncolytic Ads induce cell death through passive lysis, but their involvement in regulated cell death processes such as apoptosis or necroptosis remains unclear. Pathogenically, Ads modulate apoptosis by secreting apoptotic inhibitors/inducers, such as E4 [42] and E3 [43] for viral survival and spread. Understanding Ad[CE1A]-induced cell death mechanisms potentially aids its therapeutic success via complementary combination therapy. Ad[CE1A]-induced cell death in myeloma cells was caspase-independent; involvement of necroptosis was therefore assessed. Inhibiting necrosome complex constituents RIPK1/3 did not protect against Ad[CE1A]-induced death, but inhibiting MLKL, the final mediator of necroptosis, significantly protected myeloma cells. However, complete protection was not achieved, suggesting involvement of other mechanisms. A supporting study found Ad5-induced cell death differs from classical necroptosis, requiring MLKL but not RIPK1/3 [44]. Ad5 induced necroptosis is reported in other cancers [45, 46], therefore, Ad[CE1A] does not appear to trigger classical apoptotic or necroptotic pathways. One explanation to why complete protection against cell death following MLKL inhibition may not have been achieved with Ad[CE1A] is because other cell death pathways might be involved, such as pyroptosis and ferroptosis, which have been shown to be involved in other OV cell death mechanisms [47, 48], but these cell death pathways were not investigated as part of this study. Another explanation could be because cancer cells often have dysregulated cell death pathways, and blocking one pathway could cause Ad[CE1A] to induce cell death via another pathway, ensuring that the virus kills the host cell and spreads despite MLKL inhibition. The complexity of OV-induced cell death, which involves multiple overlapping pathways, likely accounts for why MLKL inhibition alone did not fully protect against cell death.

OV-driven ICD can initiate adaptive immune responses through DAMP/PAMP release [49]. Necroptosis/necrosis-associated cell death also results in DAMP leakage, potentially triggering anti-tumor immune responses. Ad[CE1A] significantly altered ICD markers (CALR, CD47 and ATP) in a beneficial dose and time dependent manner, aligning with Ad-driven effects observed in other cancers [49, 50]. HLA-ABC and HLA-DR expression was also significantly increased post Ad[CE1A] treatment. HLA dysregulation is recognized as a common mechanism to escape immunosurveillance [51], therefore Ad[CE1A] could potentially counteract this mechanism. Few OV studies have explored HLA expression, but a recent myeloma study found decreased HLA-ABC and HLA-DR with an oncolytic Ad (LoAD) [38], suggesting Ad[CE1A] might better counter immune dysregulation than LoAD. Further exploration of Ad[CE1A]’s immunostimulatory potential by bystander cytokine killing was undertaken. BMMC-CM from HDs and myeloma patients reduced myeloma cell viability, indicating potential bystander killing. Variable responses were observed with myeloma patients, possibly due to patient-specific immunogenic differences. These results are promising as cumulatively Ad[CE1A] could induce both anti-myeloma immune responses as well as direct myeloma killing, potentially enhancing clinical efficacy.

Results presented evidence the efficacy and safety of Ad[CE1A] in reducing myeloma tumor burden in both xenograft and syngeneic murine models. In the syngeneic model, no dose response between low and high administration was observed; however, i.p administration exhibited similar efficacy to i.v administration. This disparity might be attributed to high dose i.v Ad[CE1A] potentially triggering a stronger anti-viral immune response or take up by the liver, leading to quicker clearance than low dose i.v or high dose i.p. The syngeneic model’s validity could be debated due to Ad5’s species specificity, but multiple studies highlight Ad5’s ability to infect and replicate in murine cancer cells [52, 53]. Ad[CE1A] infection and replication efficacy was confirmed in vitro in 5TGM1 cells. The use of the human *SLAMF7* (h*SLAMF7*) promoter could also be disputed. In human myeloma cells, Ikaros zinc finger 1 (IKZF1) was identified as the pivotal transcriptional activator of *SLAMF7* [54]; however, the murine *SLAMF7* (m*SLAMF7*) promoter was found to be regulated by YY1 in B cells [55]. Since four IKZF putative binding sites are found in *mSLAMF7* promoter, it is therefore likely that IKZF-binding transactivates the *SLAMF7* promoter in human and murine cells.

Our study demonstrates for the first time that Ad[CE1A] can be enhanced by the addition of anti-myeloma drugs. Our findings reveal variations in the responses of different myeloma cell lines to combination therapy, highlighting the inherent heterogeneity within the myeloma cells. We hypothesized that the observed synergy might result from the anti-myeloma drugs enhancing viral life cycle processes. Initially, we explored whether these drugs were increasing viral infection. Proteasome inhibitors, alkylating agents and histone deacetylase inhibitors (HDACIs) have been associated with CAR upregulation in other cancers, enhancing viral infection [56–58]. Whilst our study did show increased CAR expression post anti-myeloma drugs, consistent with the literature, this did not result in significant increases in infection. This could be attributed to the high CAR expression already present in these myeloma cells, and their already efficient Ad5 infection, but this may potentially benefit cells with low CAR expression.

Our investigation into viral replication, however, highlighted that melphalan significantly increased CS1 expression, viral *E1A* expression and viral titer, indicating a clear enhancement of viral replication in myeloma cells. In contrast, for other drugs such as bortezomib, CS1 expression significantly increased, but it did not lead to a proportionate increase in *E1A* expression. The reason for the substantial increase in CS1 following bortezomib and melphalan treatment remains unknown, but a prior study observed a ~1.5-fold increase in CS1 expression post-melphalan treatment in myeloma cells [54]. In contrast, in the same study, pomalidomide significantly decreased CS1 via targeting IKZF1, a pivotal transcriptional activator of *SLAMF7* in myeloma cells [54]. Our findings only exhibited a trend of decreased CS1 expression following pomalidomide treatment. This trend was more prominent in OPM-2 cells, which have higher CS1 expression relative to other myeloma cells (Fig. 1a–c).

Notably, all anti-myeloma therapies resulted in a significant increase of viral titer, despite no increase in *E1A* levels. For bortezomib, a potential mechanism could be via HSP90 upregulation [59], which is known to be vital for Ad5 replication. Inhibition of HSP90 (by 17-AAG) correlated with decreased Ad5 replication due to decreased viral gene transcription and viral protein production [60]. Increased viral titer could also stem from prevention of proteasome degradation of Ad proteins, thereby increasing available Ad protein for transcription and virion production. In the case of panobinostat and/or pomalidomide, the mechanism could involve increased late viral gene expression, as observed in another study in glioblastoma cells post HDAC inhibition, though this increase did not result in increased viral titer [58]. These results collectively offer new insights into the complex interplay between Ad[CE1A] and anti-myeloma drugs, potentially enabling a more comprehensive therapeutic strategy against myeloma.

Myeloma patients clinically experience disease relapse due to minimal residual disease and chemotherapy resistance. Thus, investigating Ad[CE1A]‘s potential to prevent cell regrowth post-bortezomib treatment and its efficacy in bortezomib-insensitive cells was crucial. Ad[CE1A] effectively halted myeloma cell line regrowth after bortezomib treatment, while bortezomib-only treated cells recovered to vehicle levels. Notably, low Ad[CE11A] doses achieved this effect. However, in JJN-3 cells, viability began recovering at the lowest dose by day 25, possibly due to JJN-3 cell aggressiveness and lower CS1 expression relative to U266 cells. OV resistance has been reported, such as vesicular stomatitis virus resistance via APOBEC3 upregulation [61], and measles virus resistance due to strong IFIT1 expression inducing an active antiviral state [62]. Speculatively, recovery at the lowest Ad[CE1A] dose in JJN-3 cells could stem from viral resistance or a CS1-negative population. Regardless, these findings indicate Ad[CE1A]’s potential to prevent tumor regrowth and effectively target bortezomib-insensitive cells, presenting promise for relapsed/refractory disease.

## Conclusion

This work has demonstrated the potential role of Ad[CE1A] in the treatment of myeloma, and could enhance anti-myeloma responses alongside standard chemotherapies. The results presented provide a solid foundation for the development of Ad[CE1A] in combination with complementary therapies as an effective treatment for myeloma.

## Supplementary information

Supplemental Material

## Data Availability

For original data, please contact m.a.lawson@sheffield.ac.uk.
