# Supplementary material for: An oncolytic adenovirus targeting SLAMF7 demonstrates anti-myeloma efficacy
Source: Leukemia. 2025 Apr 17;39(6):1449–63. doi: 10.1038/s41375-025-02617-3 (PMC12133575; doi:10.1038/s41375-025-02617-3)
Supplement: Supplementary file 1 — Supplemental Material [file 41375_2025_2617_MOESM1_ESM.pdf]

Supplemental Data

Supplemental Methods

Cell culture

Human and murine myeloma plasma cell lines (listed in Supplemental Table 1) were maintained in complete RPMI medium[1]. The majority of assays were conducted using JJN-3, U266, OPM-2 or 5TGM1 myeloma cells, given their established status for use in *in vivo* models[2]. Bortezomib (BTZ)-resistant cell lines were maintained in the presence of BTZ as previously described[3], briefly BTZ-resistant cell lines were developed by gradually exposing JJN-3 and U266 human myeloma cell lines to increasing BTZ concentrations (0.1 nM-5 nM) in complete RPMI. Cells were treated with BTZ for 72 hours, then washed, resuspended in fresh medium to recover viability, and then re-treated at higher concentrations. BTZ resistance was confirmed with dose response curves against parental lines (Supplementary Figure 9). LNCaP, a prostate cancer cell line, used as a CS1-negative control, and HEK293A, used for the generation of Ad[CE1A] and Ad-GFP, were cultured in complete DMEM medium. Cells were routinely tested for mycoplasma, and authenticated using STR.

Primary CD138 culture and Magnetic activated cell sorting

$CD138^+$ and $CD138^-$ cells were isolated via Ficoll separation[4], and magnetic activated cell sorting (MACs) with $CD138^+$ magnetic microbeads (Miltenyi Biotech, UK). Both the $CD138^+$ and $CD138^-$ BM fractions were cultured in complete RPMI medium and complete DMEM medium respectively with 10% autologous serum.

Generation of Ad[CE1A]

The Recombinant *SLAMF7*-specific replication-competent adenovirus (Ad[CE1A]), in which the promoter element of the *SLAMF7* gene drives the expression of the *E1A* gene was constructed using the E1/E3-deleted AdEasy system according to manufacturer's instructions (Agilent, UK). Briefly, the

CS1 promoter sequence was isolated from a CS1 Luc reporting plasmid (SwitchGear, Belgium) using KpnI and HindIII and ligated into pShuttle using the same enzymes. In order to place E1A under the control of the CS1 promoter, the pShuttle-CS1 plasmid was digested with SalI and the E1A gene was PCR amplified using primers. The digested plasmids and PCR products were ligated together, and the insert checked for orientation using a digest with AhdI and XbaI. The pShuttle-CS1:E1A combined with the pAdeasy vector. The recombined vector was transfected into HEK293A cells (a suitable cell line for virus propagation) and the virus isolated using Adeno-X Maxi Purification Kit (Clonetech, France). All Viruses used were amplified in HEK293A cells, purified by caesium chloride gradient ultracentrifugation and titred using the Adeno-X^{TM} rapid titre kit (Clontech, UK).

Assessment of adenoviral infection

1x10^5 cells were infected with Ad-GFP MOI 2 or 20 or PBS control. After 24 and 48 hours, GFP expression was qualitatively visualised using fluorescent microscopy (EVOS^{TM} FL Auto), then quantified using flow cytometry.

Flow cytometry

Cell surface and/or intracellular protein expression was analysed in cells treated ± Ad[CE1A] ± anti-myeloma therapies (doses as indicated). After indicated time points, cells were incubated with fluorescently conjugated antibodies or dose-matched isotype control for 45 minutes at 4°C in the dark. For intracellular proteins, cells were permeabilized first and then stained with corresponding antibodies. PI or TO-PRO-3 was used prior to flow cytometry to identify and exclude dead cells.

Myeloma patient bone marrow mononuclear cells (BMMCs) were collected from BM aspirates, treated with Ad[CE1A] (MOI: 30 or PBS) for 72 hours, and then populations of T cells (CD4 and CD8)

and NK cells were determined by flow cytometry using fluorescence minus one controls for gating. Data were analysed by a One-way ANOVA.

BMMC-conditioned medium

$CD138^-$ BMMCs from myeloma patients or healthy donors were seeded at $2x10^6$ cells/ml and treated with Ad[CE1A] MOI 20 or vehicle PBS. After 48 hours, conditioned media (CM) was collected by removing cells via centrifugation (400g x 5 minutes) which was then sterile filtered using a 0.2 µm syringe filter and Ad[CE1A] virions were UV inactivated using a UV crosslinker. CM was UV treated for 1 hour in 500 µL aliquots in an open 24 well plate in a cell culture hood. UV inactivation was confirmed by viral titre assays.

Bystander Cytokine Killing

$1x10^4$ JJN-3, U266 and OPM-2 cells were seeded per well into 96 well plates in 50 µL of complete RPMI media. Cells were treated with 50 µL of UV-inactivated control or Ad[CE1A] CM from BMMCs from HDs or myeloma patients. Cells were seeded in quadruplicate. After 96 hours, cell viability was assessed by AlamarBlue® assay.

Apoptosis assays

$1x10^5$ cells were seeded in triplicate in 500 µL of complete RPMI media and treated with Ad[CE1A] MOI 20. After 6 and 24 hours, cells were collected, and apoptosis was assessed by staining cells with Annexin V conjugated to FITC according to manufacturer's instructions (BD Biosciences, UK), TOPRO-3 was added and analysed by flow cytometry.

Flow cytometric and Histological assessment of tumour burden

BM was flushed from a femur with PBS. After lysing red blood cells, BM was divided in two and stained with an anti-HLA-FITC or a dose matched isotype control. Statistical outliers were determined following Grubb's test (alpha 0·05). One mouse was determined to be an outlier in the Ad[CE1A] treatment group and data removed. Tibiae were fixed in 10% Formalin for 48 hours, then stored in 70% ethanol. After decalcification, tibiae were paraffin-embedded, and 3 µM longitudinal sections were immunohistochemically assessed using a polyclonal IgG Kappa light chain antibody (proteintech; 14678-1-AP, 1:300).

Micro-CT

Ex vivo micro-CT was performed in the tibia of mice using a SkyScan 1272 micro-CT scanner (Bruker) at 4.3 µm voxel size, 0.5 mm aluminium filter, 50 KV, 200µA for a 180 degrees scan with 0.7 degrees rotation step, and image reconstruction was performed using SkyScan software NRecon (1.6.9.4). Trabecular analysis was performed using CTAn (1.8.1.2, Bruker). Tibial trabecular bone was analysed in a 1 mm region with a 0.2 mm and 0.4 mm distal offset from the growth plate, respectively.

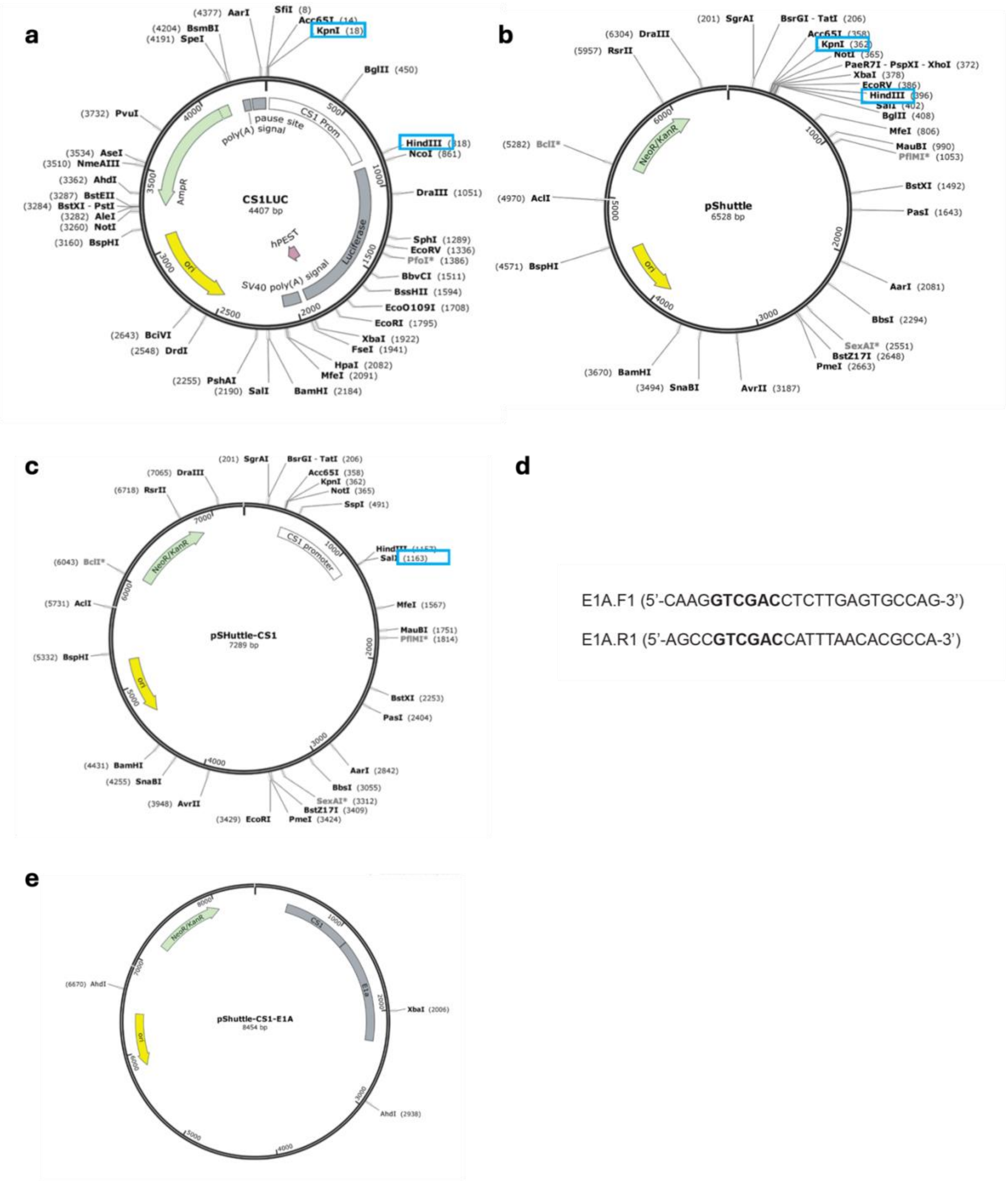

Supplemental Figure 1: **Plasmid schematics used in the construction of Ad[CE1A]. (a)** Plasmid map of CS1:Luc reporting plasmid with restriction enzymes (KpnI and HindIII) used to cut out the CS1 promoter. **(b)** Plasmid map of the destination vector pShuttle showing the same enzymes. **(c)** Plasmid map of pShuttle-CS1 following ligation showing locations of the SalI restriction site used to insert amplified E1A. **(d)** E1A primer for PCR amplification. **(e)** Plasmid map of complete pShuttle-CS1-E1A.

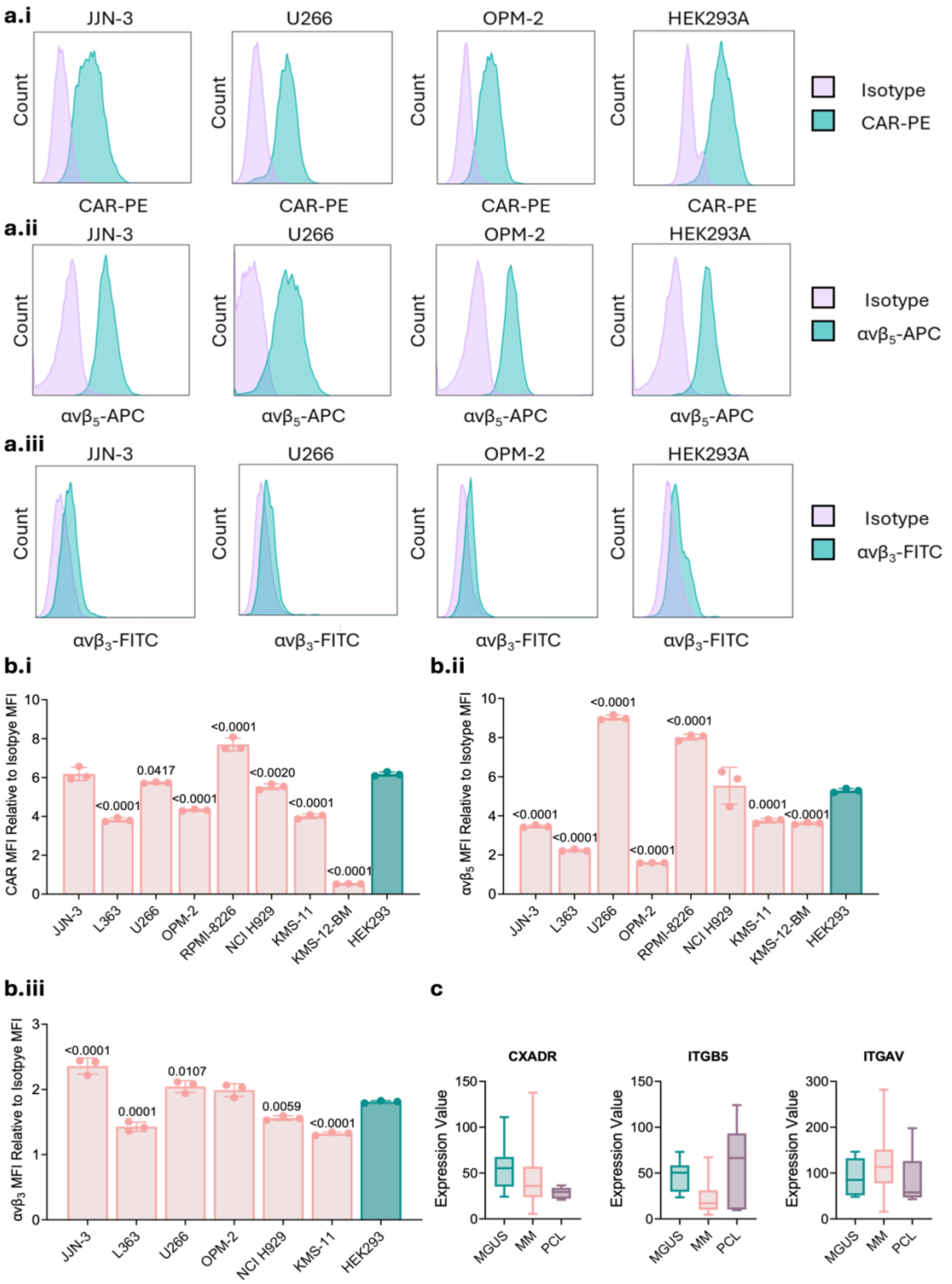

Supplemental Figure 2: Expression levels of CAR, $\alpha v\beta_5$ and $\alpha v\beta_3$ in myeloma cell lines and patient cells. Flow cytometry histograms of **(a.i)** coxsackie adenovirus receptor (CAR), **(a.ii)** $\alpha v\beta_5$ and **(a.iii)** $\alpha v\beta_3$ mean fluorescent intensity (MFI) by JJN3, U266, OMP-2 and HEK293A cells. Comparison of MFI relative to isotype for **(b.i)** , **(b.ii)** $\alpha v\beta_5$ and **(b.iii)** $\alpha v\beta_3$ in myeloma cell lines vs. HEK293A cells. n=3 biological replicates ±SD. p values: one-way ANOVA Dunnett's correction. **(c)** Gene expression of CXADR (CAR), ITGB5 (integrin subunit β) and ITGAV (integrin subunit α) from purified plasma cells from MGUS (n=7), myeloma (n=39) and PCL (n=6) patients.

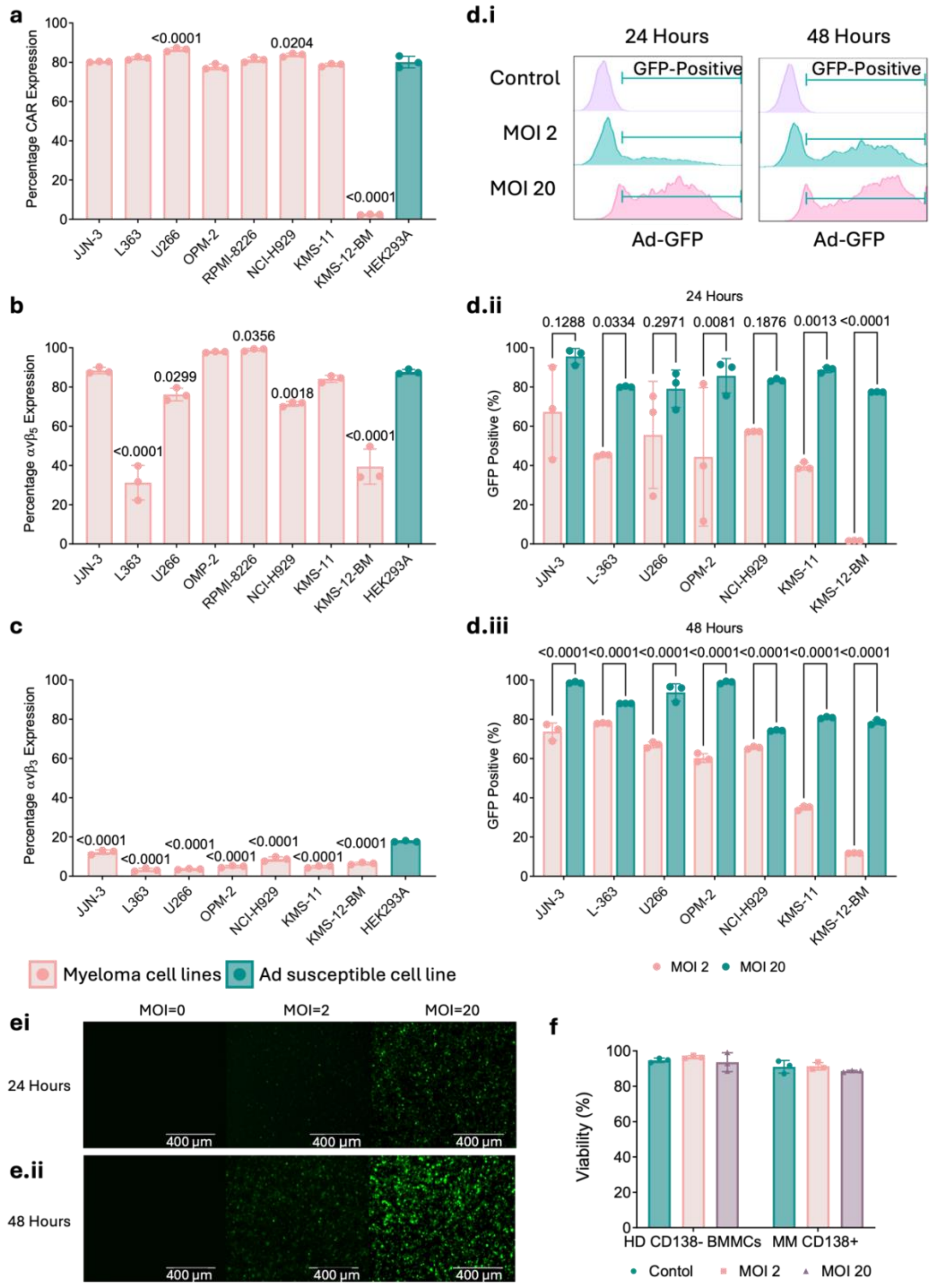

Supplemental Figure 3: Human myeloma cell lines are susceptible to adenovirus infection. Percentage expression of **(a)** coxsackie adenovirus receptor (CAR), **(b)** $\alpha v\beta_5$ integrin and **(c)** $\alpha v\beta_3$ in human myeloma cell lines vs. HEK293A cells. n=3 biological replicates ±SD. p values: one-way ANOVA vs. HEK293A cells. **(d.i)** Representative mean fluorescent intensity histograms of Ad-GFP expression (MOI 2 or MOI 20) in human myeloma cell lines at 24 and 48 hours by flow cytometry. Percentage of GFP-positive cells at **(d.ii)** 24 and **(d.iii)** 48 hours. n=3 biological replicates ±SD. p values: 2-way ANOVA Šidák's correction. Representative images using live fluorescent microscopy of Ad-GFP (MOI 2 or 20) in the JJN-3 human myeloma cell line at **(e.i)** 24 and **(e.ii)** 48 hours. **(f)** Cell viability of CD138-positive plasma cells from myeloma patients and CD138-negative BMMCs from healthy donors infected with Ad-GFP (MOI 2 or 20) compared to vehicle control. n=3 ±SD.

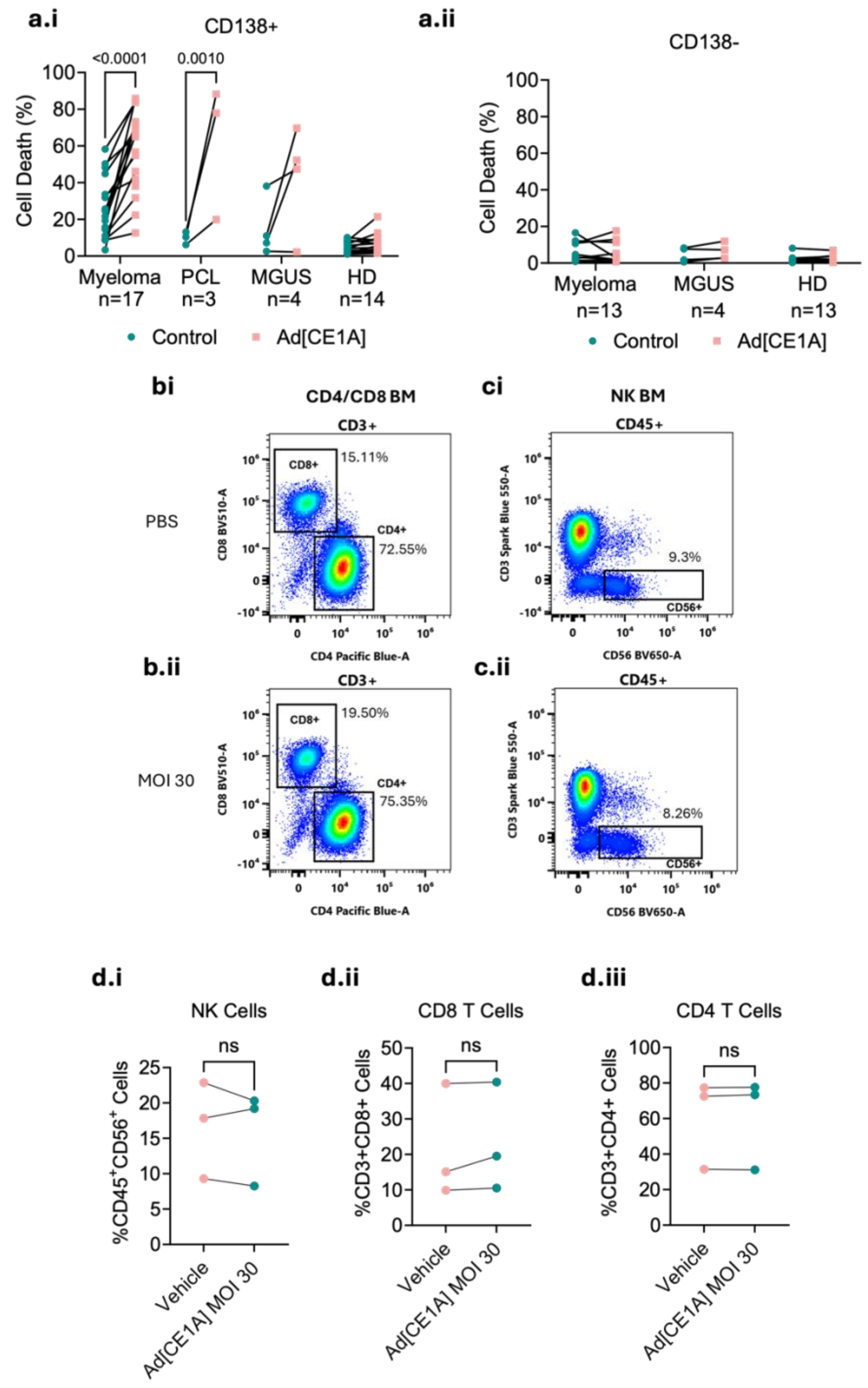

Supplemental Figure 4: Ad[CE1A] causes oncolysis in primary patient derived myeloma cells, but does not cause oncolysis in primary patient bone marrow NK, CD8^{+}, or CD4^{+} T cell populations. (a.i) Ad[CE1A] cytotoxicity (MOI 20) after 96 hours in myeloma (MM) (n=17), plasma cell leukemia (PCL) (n=3), monoclonal gammopathy of undetermined significance (MGUS) (n=4) and healthy donors (HD) CD138^{+} cells (n=14). **(a.ii)** Ad[CE1A] cytotoxicity (MOI 20) after 96 hours in MM (n=13), MGUS (n=4) and HD (n=13) CD138^{-} cells. p values: paired T test. Bone marrow mononuclear cells (BMMCs) from newly diagnosed MM patients were treated with vehicle or Ad[CE1A] at MOI 30. After 72 hours, cells were stained for CD3^{+}CD4^{+} T cells, CD3^{+}CD8^{+} T cells, and CD45^{+}CD56^{+} NK cells, and analyzed by flow cytometry. Representative dot plots show CD3^{+}CD4^{+} and CD3^{+}CD8^{+} T cells with **(b.i)** vehicle and **(b.ii)** Ad[CE1A] treatment, and CD45^{+}CD56^{+} NK cells with **(c.i)** vehicle and **(c.ii)** Ad[CE1A]. Quantification of **(d.i)** NK cells, **(d.ii)** CD8^{+} T cells, and **(d.iii)** CD4^{+} T cells; n=3, paired T test.

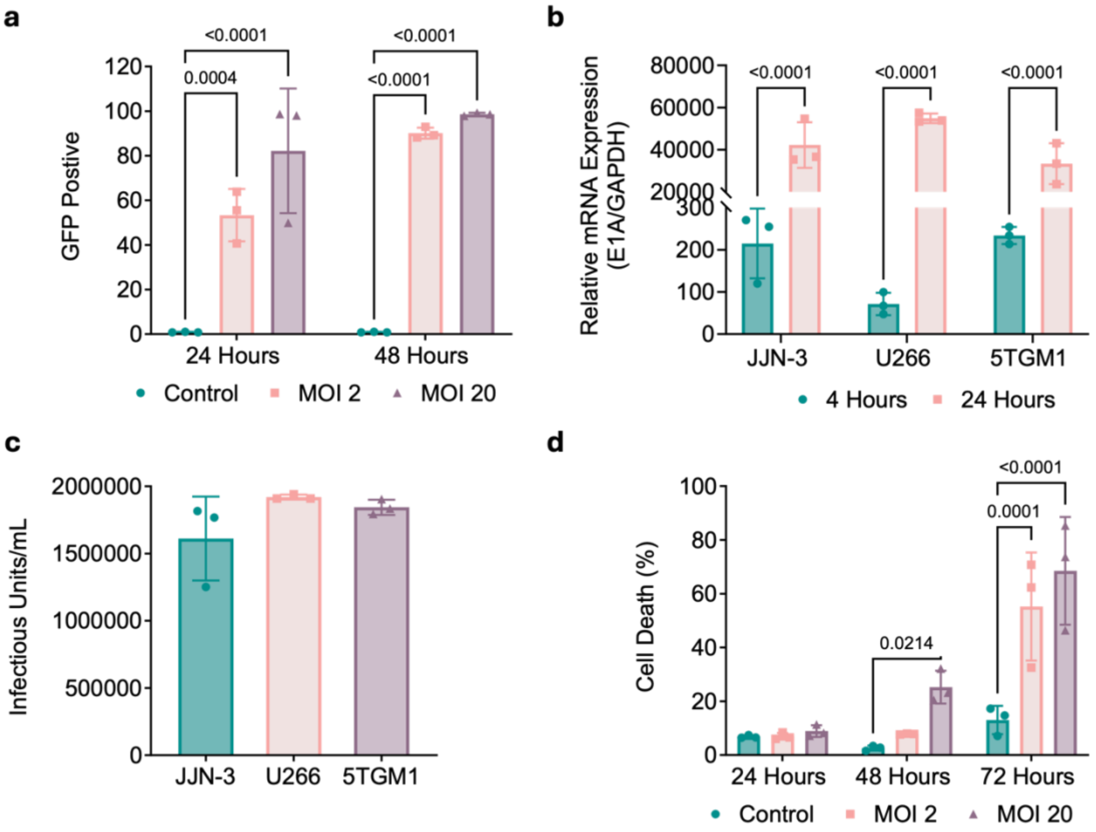

Supplemental Figure 5: Murine myeloma cell line, 5TGM1, is susceptible to Ad[CE1A] replication and oncolysis. (a) Percentage of GFP-positive 5TGM1 cells following infection with Ad-GFP (MOI 2 or 20) at 24 or 48 hours compared to control. n=3 biological replicates ±SD. p values: 2-way ANOVA Šidák's correction. **(b)** *E1A* mRNA expression in murine 5TGM1 and human JJN-3 and U266 myeloma cell lines at 4 and 24 hours post Ad[CE1A] infection (MOI 20) compared to vehicle controls n=3 biological replicates ±SD. p values: 2-way ANOVA Šidák's correction. **(c)** Infectious virion production in murine 5TGM1 and human JJN-3 and U266 myeloma cells after 72 hours using Adeno-X™ rapid titre kit. n=3 biological replicates ±SD. **(d)** Dose and time response of Ad[CE1A] cytotoxicity in 5TGM1 cells assessed by PI staining and flow cytometry. n=3 biological replicates ±SD. p values: 2-way ANOVA Dunnett's correction.

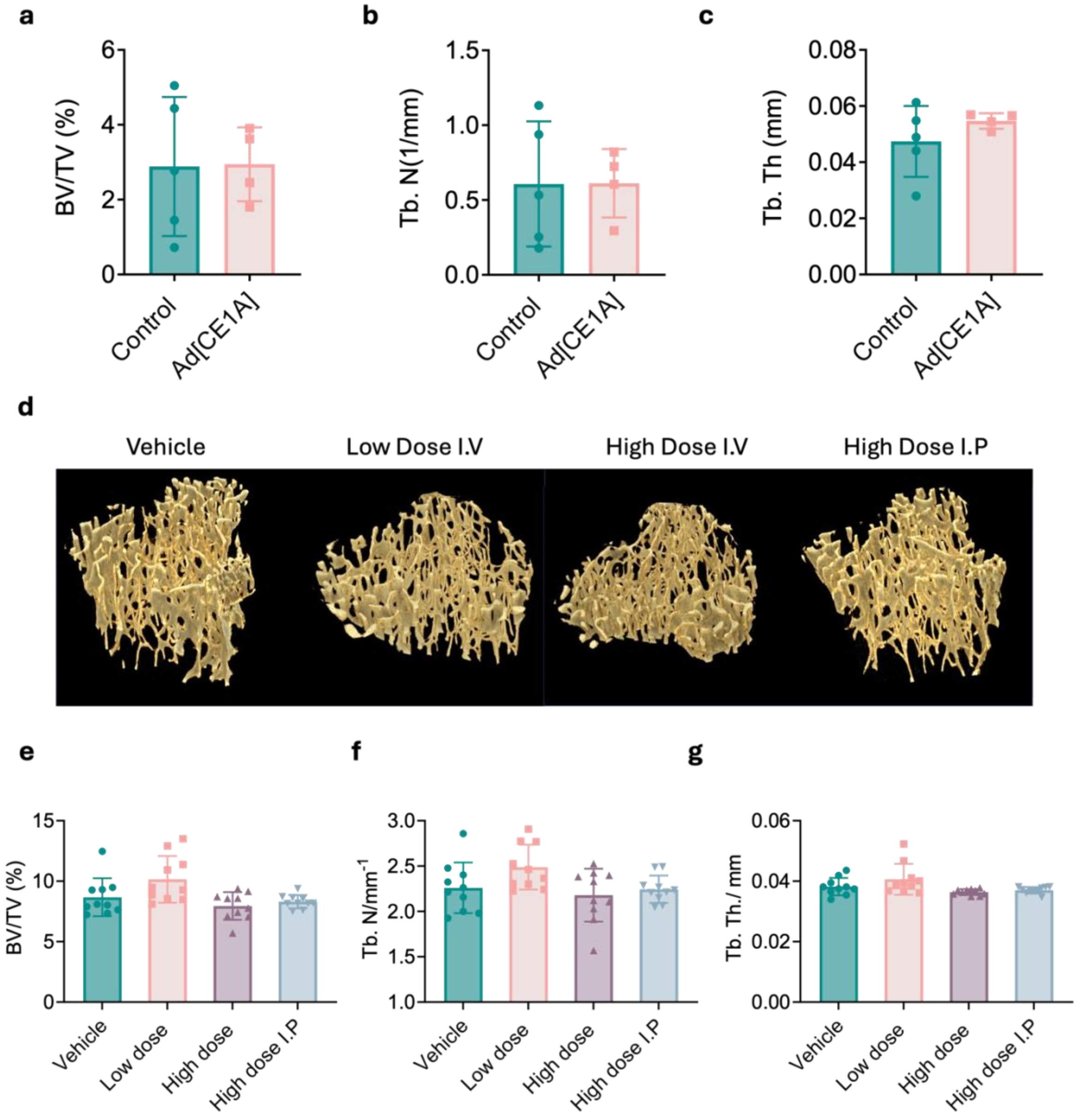

Supplemental Figure 6: Ad[CE1A] has no negative effects on myeloma bone disease. Xenograft experimental plan: Female NSG mice were inoculated with 1x10^6 U266 (I.V). 5 weeks after tumor development mice were randomised into vehicle (PBS) (n=5) or Ad[CE1A] (10^7 ifu) (n=4) (I.V) 2x/week for 3 weeks. After 3 weeks, mice were euthanized. **(a)** Trabecular bone volume/tissue volume (BV/TV) **(b)** trabecular number (Tb. N) and **(c)** trabecular thickness (Tb. Th) were analysed by micro-CT *ex vivo* in the tibias. **Syngeneic experimental plan:** Male C57BL/KaLwRij mice were inoculated with 2x10^6 5TGM1-Luc cells (I.V). After three days, mice were randomized into vehicle (PBS), 10^7 ifu of Ad[CE1A] I.V (low dose group), 10^8 ifu of Ad[CE1A] I.V (high dose group) and 10^8 ifu of Ad[CE1A] I.P (n=10/group). Treatment was administered 2x/week for 4 weeks. After 28 days, mice were euthanized. **(d)** Representative 3D trabecular bone micro-CT images from the tibias of 5TGM1 mice treated with either vehicle, Ad[CE1A] low dose I.V, Ad[CE1A] high dose I.V or Ad[CE1A] high dose I.P. **(e)** BV/TV, **(f)** Tb. N and **(g)** Tb. Th were analysed by micro-CT *ex vivo* in the tibias.

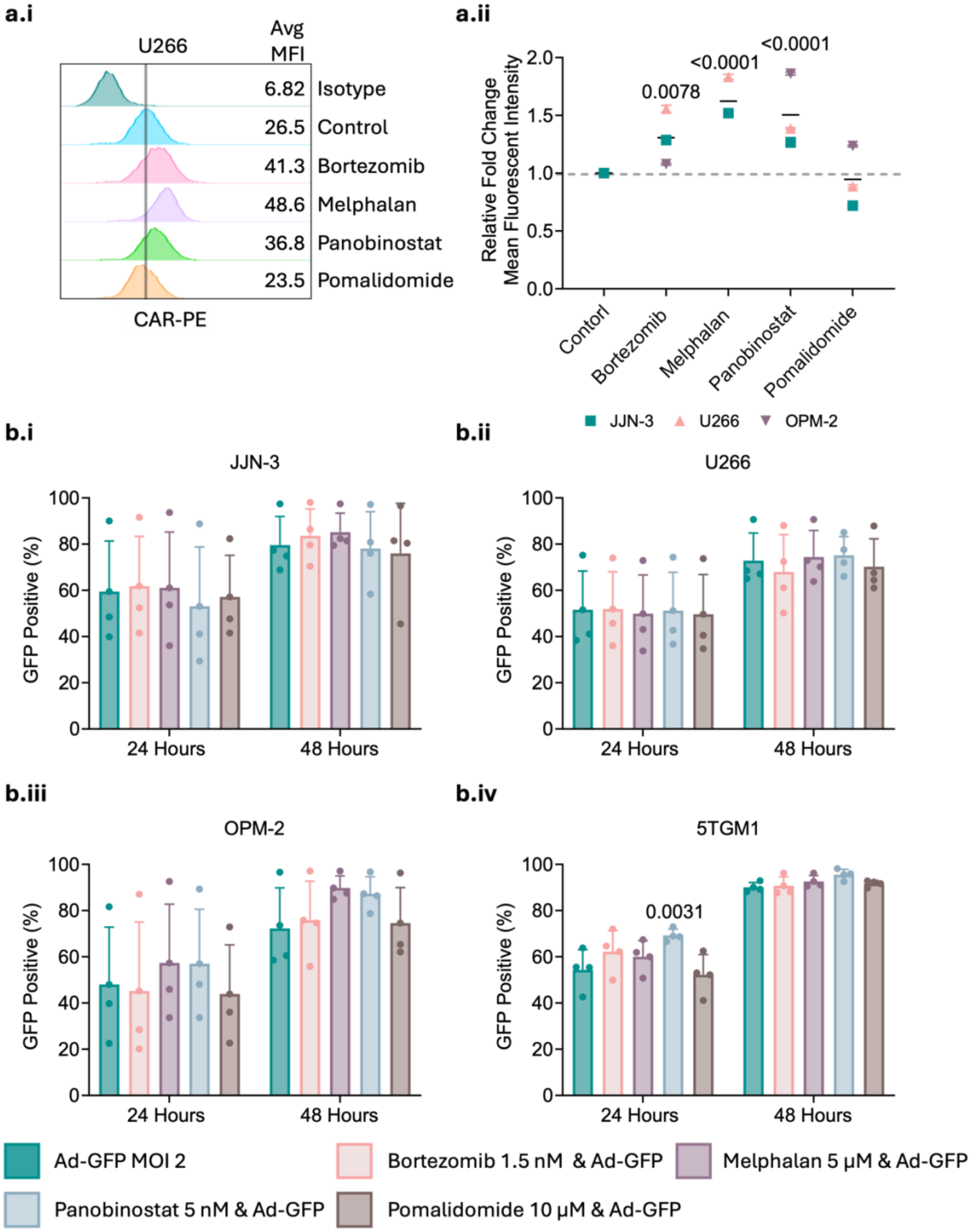

Supplemental Figure 7: CAR expression is enhanced with addition of anti-myeloma drugs, but this does not impact initial viral infection. CAR expression after 48 hours anti-myeloma treatment. **(a.i)** Representative histogram of CAR MFI after 48 hour anti-myeloma treatment in U266 cells. **(a.ii)** Average relative fold change of CAR MFI post anti-myeloma drugs vs untreated control in JJN-3, U266 and OPM-2 cells. Black bars indicate the means of the cell lines. n=3 biological replicates ±SD. p values: one-way ANOVA Dunnett's correction. Ad-GFP (MOI 2) expression percentage ± anti-myeloma drugs vs. Ad-GFP alone in **(b.i)** JJN-3, **(b.ii)** U266, **(b.iii)** OMP-2 and **(b.iv)** 5TGM1 cells after 24 and 48 hours. n=4 biological replicates ±SD. p values: 2-way ANOVA Dunnett's correction.

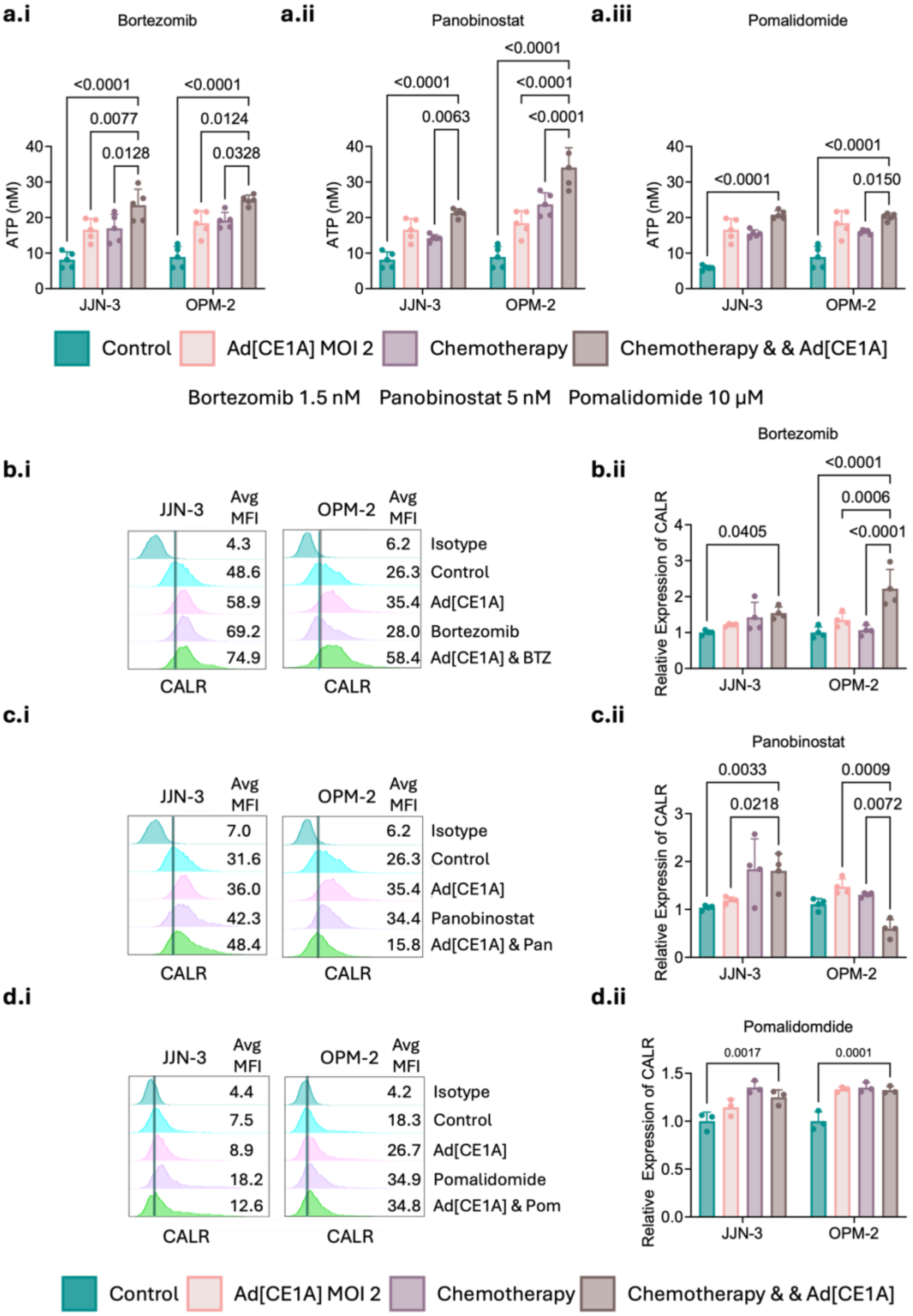

Supplemental Figure 8: Enhanced immunogenic cell death markers by Ad[CE1A] in combination with anti-myeloma drugs. JJN-3, and OPM-2 cells treated with Ad[CE1A] MOI 2 ± anti-myeloma chemotherapies, **(a.i)** BTZ 1.5 nM, **(a.ii)** Pan 5 nM and **(a.iii)** Pom 10 µM. After 24 hours supernatant was collected, and ATP concentration was determined by the ENLITEN® ATP assay using an ATP standard curve. n=3 biological replicates ±SD. p values: one-way ANOVA Tukey's correction. Representative histograms of cell surface CALR MFI in viable JJN-3, and OPM-2 cells treated with Ad[CE1A] MOI 2 ± anti-myeloma chemotherapies, **(b.i)** BTZ 1.5 nM, , **(c.i)** Pan 5 nM and **(d.i)** Pom 10 µM after 24 hours. Relative fold change of cell surface CALR MFI in JJN-3 and OPM-2 cells treated with Ad[CE1A] MOI 2 ± anti-myeloma chemotherapies, **(b.ii)** BTZ 1.5 nM, **(c.ii)** Pan 5 nM and **(d.ii)** Pom 10 µM after 24 hours. n=4 biological replicates ±SD. p values: two-way ANOVA Tukey's correction.

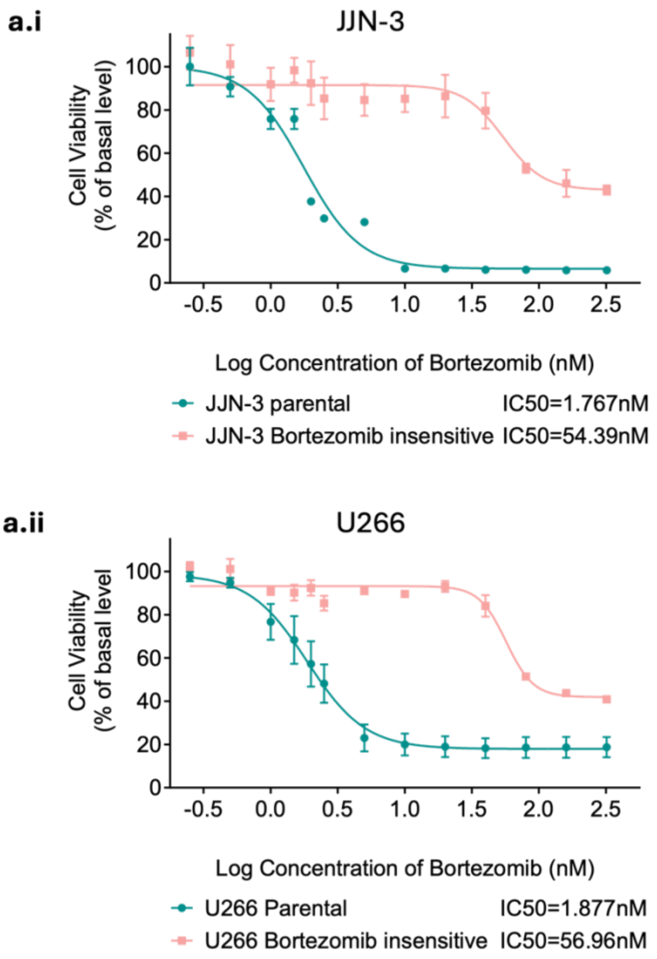

Supplemental Figure 9: Bortezomib dose response curves in bortezomib-insensitive and parental myeloma cell lines. (a.i) JJN-3 parental and bortezomib-insensitive cells and **(a.ii)** U266 parental and bortezomib-insensitive cells were treated with a log dose response concentration of bortezomib. After 72 hours, an AlamarBlue® assay was performed and dose response curves generated.

Supplemental Table 1: Cell line origins and characteristics[5,6].

Cell line	Origin	Cell Surface Markers	Cytogenetics	Purchased/ Supplied From
JJN-3	PCL in 57-year-old female, IgAκ. Established from BM.	$CD138^+$ $HLA\text{-}DR^+$	Hypotriploid 58-67, XX t(14;16) 9% polyploidy	DSMZ, Germany
L-363	PCL in 36-year-old female, IgG. Established from peripheral blood.	CD138 $^+$ HLA-DR $^+$	Hyperdiploid 11% polyploidy Del(17)(p12) 11q13, 14q32	Dr Khanim, University of Birmingham UK
OPM-2	PCL in 56-year-old female, IgGλ. Established from peripheral blood.	$CD138^+$ $CD38^+$ $HLA\text{-}DR^-$	Hypertriploid/ hypotetraploid 77-82, XX t(4;14)*	DSMZ, Germany
U266	PCL in 53-year-old male, IgEλ. Established from peripheral blood.	$CD38^+$ $CD138^+$ $HLA\text{-}DR^+$	Hypodiploid 6.5% polyploidy t(11;14) 11q13	LGC Standards, UK
RMPI-8226	PCL in 61-year-old male, IgGλ. Established from peripheral blood.	$CD38^+$ $CD138^+$ $HLA\text{-}DR^+$	Hypotriploid 7.5% polyploidy 62-67, XXY t(14;16)	DSMZ, Germany
NCI-H929	MM in 62-year-old Caucasian female, IgAκ. Established from pleural effusion.	$CD38^+$ $CD138^+$ $HLA\text{-}DR^-$	Hypodiploid 16% polyploidy 43-46, X t(4;14)*	Dr Khanim, University of Birmingham UK
KMS-11	MM in 67-year-old female, IgGκ. Established from a pleural effusion.	$CD138^+$	t(4;14), t(14;16) hypertriploid	DSMZ, Germany
KMS-12-BM	MM in 64-year-old women, non-secretory. Established from the BM.	$CD38^+$ $CD138_+$ $HLA\text{-}DR^-$	Hypertriploid 3% polyploidy t(11;14)	DSMZ, Germany
5TGM1	Established from inbred C57BL/KaLwRij mice that spontaneously developed MM	$CD138^+$	t(14 ;16)	See acknowledgements
LNCaP	Isolated from the lymph node of a 50-year-old prostate cancer patient	-	-	ATCC, USA
HEK293A	Isolated from a female human embryonic kidney	-	-	ATCC, USA

PCL: Plasma Cell Leukemia.

Supplemental Table 2: Patient information.

ID	Diagnosis	Age	Sex	Subtype	MC size (g/L)	LC ratio	Hb (g/L)	Creatinine (mg/dL)	eGFR ml/min/1.73m²	Calcium (mg/dL)	Plasma cell (%)
PCL1	PCL	70	M	ND	ND	ND	ND	ND	ND	ND	Unknown
PCL2	PCL	75	M	IgGκ	19.2	ND	125	108	58	2.3	40
PCL3	PCL	70	F	FLCκ	ND	ND	ND	ND	ND	ND	50
MM1	MM	87	M	IgG	15.3	ND	78	164	32	2.6	73
MM2	MM	75	M	IgGκ	19.8	ND	110	74	85	2.3	15
MM3	MM	70	M	IgGκ	12.5	10.9	104	151	40	2.5	12
MM4	MM	51	M	IgGλ	14.1	ND	139	ND	ND	ND	50
MM5	MM	68	M	IgAκ	15.9	2.9	143	95	70	2.4	14
MM6	MM	78	F	FLCλ	ND	<0.1	97	117	39	2.4	50
MM7	MM	67	F	IgGκ	31.9	5.3	127	51	>90	2.4	13
MM8	MM	68	M	IgGλ	38.4	ND	103	88	77	2.4	90
MM9	MM	76	F	IgGλ	28.8	<0.1	98	147	30	2.5	16
MM10	MM	74	M	IgGκ	20.5	ND	105	124	49	2.3	32
MM11	MM	86	M	IgGκ	46.4	ND	92	64	84	2.6	70
MM12	MM	30	M	IgGκ	27.4	ND	120	90	82	2.4	30
MM13	MM	67	M	IgGλ	15.4	ND	130	80	88	2.3	10
MM14	MM	74	M	IgGκ	49.2	ND	90	80	84	2.7	65
MM15	MM	70	F	IgGλ	32.1	<0.1	93	96	52	2.5	70
MM16	MM	64	F	FLCλ	2.1	<0.1	124	65	86	2.3	34
MM17	MM	76	F	IgAκ	21.8	ND	123	82	60	2.47	42
MGUS1	MGUS	79	F	IgGκ	11.4	ND	101	112	40	2.4	8
MGUS2	MGUS	55	M	IgGκ	14	2.5	149	85	88	2.3	Unknown
MGUS3	MGUS	71	F	FLCκ	5	50.7	129	92	54	2.3	6
MGUS4	MGUS	80	F	IgGκ	2.4	1.4	96	122	36	2.4	3
HD1-14	HD	35-60	M/F	ND	ND	ND	ND	ND	ND	ND	Unknown
BMMC1	MM	67	M	IgGλ	6.5	<0.1	156	79	88	2.3	25
BMMC2	MM	66	M	IgGκ	11.1	63.3	117	92	74	2.4	75
BMMC3	MM	76	F	IgGκ	8.3	2.8	126	47	>90	2.2	15

PCL = plasma cell leukemia, MM = multiple myeloma, MGUS = monoclonal gammopathy of undetermined significance, HD = Healthy donors, BMMC = bone marrow mononuclear cells, M = male, F = Female, κ = kappa, λ = lambda, MC = Monoclone, FLC =Free light chain, Hb = Haemoglobin, eGFR = estimated glomerular filtration rate, ND = not determined.

Supplemental Table 3: Antibodies used in flow cytometry.

Antibody Specificity	Clone	Host	Fluorochrome	Cell Density & Amount of Antibody Used	Supplier
Human Anti-CAR	RmcB	Mouse	PE	$5x10^5$ cells 1 µL used.	Merk Millipore
Human CAR Isotype	MOPC-21	Mouse	PE	$5x10^5$ cells 2.5 µL used.	Biolegend
Human Anti-αvβ3	23C6	Mouse	FITC	$5x10^5$ cells 2.5 µL used.	Biolegend
Human Anti-$\alpha v\beta_3$ isotype	MOPC-21	Mouse	FITC	$5x10^5$ cells 2.5 µL used.	Biolegend
Human Anti-$\alpha v\beta_5$	NKI-M9	Mouse	APC	$5x10^5$ cells 2.5 µL used.	Biolegend
Human Anti-$\alpha v\beta_5$ isotype	MOPC-173	Mouse	APC	$5x10^5$ cells 2.5 µL used.	Biolegend
Human Anti-CD47	CC2C6	Mouse	APC	$2.5x10^5$ cells 2.5 µL used.	Biolegend
Human Anti-CD47 Isotype	MOPC-21	Mouse	APC	$2.5x10^5$ cells 2.5 µL used.	Biolegend
Human Anti-CD3	SK7	Mouse	Spark Blue™ 550	$5x10^5$ cells 2.5 µL used.	Biolegend
Human Anti-CD45RA	HI100	Mouse	Alexa Fluor® 700	$5x10^5$ cells 2.5 µL used.	Biolegend
Human Anti-CD4	OKT4	Mouse	Pacific Blue™	$5x10^5$ cells 2.5 µL used.	Biolegend
Human Anti-CD8	SK1	Mouse	Brilliant Violet 510™	$5x10^5$ cells 2.5 µL used.	Biolegend
Human Anti-CD56	HCD56	Mouse	Brilliant Violet 650™	$5x10^5$ cells 2.5 µL used.	Biolegend
Polyclonal calreticulin	PA3-900	Rabbit	N/A	$2.5x10^5$ cells 1 µL used.	Invitrogen
Calreticulin Isotype	Poly29108	Rabbit	N/A	$2.5x10^5$ cells 10 µL used.	Biolegned
Calreticulin Secondary	Poly4064	Donkey	FITC	$2.5x10^5$ cells 10 µL used.	Biolegend
Anti-human HLA-ABC	W6/32	Mouse	APC	$2.5x10^5$ cells 2.5 µL used.	Biolegend
Human Anti-HLA-ABC isotype	MOPC-173	Mouse	APC	$5x10^5$ cells 2.5 µL used.	Biolegend
Anti-human HLA-DR	LN3	Mouse	APC	$5x10^5$ cells 2.5 µL used.	Biolegend
Anti-human HLA-DR isotype	MG2b-57	Mouse	APC	$5x10^5$ cells 2.5 µL used.	Biolegend

Supplemental Table 4: Primers used for RT-qPCR.

SYBR® Green	
**Gene**	**Sequence**
Human GAPDH	F 5’-TGCACCACCAACTGCTTAGC<br>R 5'-GGCATGGACTGTGGTCATGAG
E1A	F 5'-ATGGGCAGTCGGTGATAGAGT<br>R 5'-CTCAGGCTCAGGTTCAGAC
Human Caspase 3	F’ 5’-AAAGCACTGGAATGACATC<br>R 5’-CGCATCAATTCCACAATTTC
Human Caspase 8	F 5’-CTACAGGGTCATGCTCTATC<br>R 5’-ATTTGGAGATTTCCTCTTGC
Human Caspase 9	F 5’CCTACTCTACTTTCCCAGGTTTT<br>R 5’-GTGAGCCCACTGCTCAAAGAT
Human BCL2	F’ 5’-GGAAGTGAACATTTCGGTGAC<br>R 5’-GCCTCTCCTCACGTTCCC
Human Fas Ligand	F 5’-ATCCCTCTGGAATGGGAAGA<br>R 5’-CCATATCTGTCCAGTAGTGC
Human Bax	F 5’-CAAACTGGTGCTCAAGGCC<br>R 5’-GCACTCCCGCCACAAAGAT
Human BID	F 5’-GAGGATTGTGGCCTTCTTTG<br>R 5’-CTCAGCCCAGACTCACATCA
**Taqman™**	
**Gene**	**Assay ID**
Human GAPDH	Hs00266705_g1 (ThermoFisher Scientific)
Human SLAMF7	Hs00904275_m1 (ThermoFisher Scientific)

Supplemental References

1. Lawson MA, Paton-Hough JM, Evans HR, Walker RE, Harris W, Ratnabalan D et al. NOD/SCID-GAMMA mice are an ideal strain to assess the efficacy of therapeutic agents used in the treatment of myeloma bone disease. *PLoS One*. 2015;10(3). doi:10.1371/journal.pone.0119546

2. Paton-Hough J, Chantry AD, Lawson MA. A review of current murine models of multiple myeloma used to assess the efficacy of therapeutic agents on tumour growth and bone disease. *Bone*. 2015;77:57-68. doi:10.1016/j.bone.2015.04.004

3. McDermott M, Eustace AJ, Busschots S, Breen L, Crown J, Clynes M et al. In vitro Development of Chemotherapy and Targeted Therapy Drug-Resistant Cancer Cell Lines: A Practical Guide with Case Studies. *Front Oncol*. 2014;4. doi:10.3389/FONC.2014.00040

4. Muthana M, Giannoudis A, Scott SD, Fang HY, Coffelt SB, Morrow FJ et al. Use of macrophages to target therapeutic adenovirus to human prostate tumors. *Cancer Res*. 2011;71(5):1805-1815. doi:10.1158/0008-5472.CAN-10-2349

5. Gooding RP, Bybee A, Cooke F, Little SG, Marsh E, Coelho E et al. Phenotypic and molecular analysis of six human cell lines derived from patients with plasma cell dyscrasia. *Br J Haematol*. 1999;106(3):669-681. doi:10.1046/J.1365-2141.1999.01602.X

6. Lombardi L, Poretti G, Mattioli M, Fabris S, Agnelli L, Bicciato et al. Molecular characterization of human multiple myeloma cell lines by integrative genomics: Insights into the biology of the disease. *Genes, Chromosome Cancer*. 2007;46(3):226-238. doi:10.1002/GCC.20404
